# Autoantibody repertoire analysis in paraneoplastic pemphigus reveals novel targets linked to mucocutaneous blistering and bronchiolitis obliterans

**DOI:** 10.1038/s43856-025-01335-2

**Published:** 2026-01-10

**Authors:** Daniel Eriksson, Maribel Aranda-Guillén, Norito Ishii, Axel Cederholm, Anish Behere, Fahad Ahmed, Juliaana Katto, Sara Öster, Helen Kaipe, Dhifaf Sarhan, Olle Kämpe, Takashi Hashimoto, Nils Landegren

**Affiliations:** 1 Department of Immunology, Genetics and Pathology, Uppsala University, Uppsala, Sweden; 2 Department of Medicine (Solna), Center for Molecular Medicine, Karolinska Institutet, Stockholm, Sweden; 3 Department of Dermatology, Kurume University School of Medicine, Kurume, Japan; 4 Science for Life Laboratory, Department of Medical Biochemistry and Microbiology, Uppsala University, Uppsala, Sweden; 5 Department of Laboratory Medicine, Division of Immunology, Karolinska Institutet, Stockholm, Sweden; 6 Department of Immunology and Transfusion Medicine, Karolinska University Hospital, Stockholm, Sweden; 7 Department of Laboratory Medicine, Division of Pathology, Karolinska Institutet, Stockholm, Sweden; 8 Department of Endocrinology, Karolinska University Hospital, Stockholm, Sweden; 9 Department of Dermatology, School of Medicine, Osaka Metropolitan University, Osaka, Japan

**Keywords:** Diagnostic markers, Autoimmune diseases

## Abstract

**Background:**

Paraneoplastic autoimmunity develops as consequences of immune reactions to cancer and exhibits a wide range of clinical manifestations. The autoimmune signs are often visible before the underlying malignancy is diagnosed, and a prompt diagnosis of paraneoplasia is crucial to enable early tumor detection. We characterized the immune responses underlying the severe mucocutaneous blistering disease paraneoplastic pemphigus.

**Methods:**

We used a two-step approach to proteome-wide autoantibody repertoire analysis and independent validation in patients with paraneoplastic pemphigus (n = 84) and non-paraneoplastic autoimmune blistering diseases (n = 103).

**Results:**

Our findings reveal that paraneoplastic pemphigus features a broad repertoire of disease-specific autoantibodies that mainly target tissue-specific proteins in the skin and mucous membranes. Importantly, we identify SERPINB3 as a major autoantibody target with an expression pattern and clinical association suggesting a role in bronchiolitis obliterans. Autoantibody profiles are similar across neoplasias, except in thymoma patients, who additionally express multiple cytokine autoantibodies.

**Conclusions:**

Our findings reveal a disease-defining autoantibody repertoire in paraneoplastic pemphigus that corresponds with clinical manifestations and holds high potential for early cancer detection in patients with blistering disease.

## Plain language summary

When the immune system reacts against cancer cells, it can sometimes mistakenly attack self-tissues in a process called autoimmunity. In some patients, these autoimmune symptoms present even before the cancer is detected, making early diagnosis of cancer-associated autoimmunity very important. In this study, we characterized the proteins targeted by the immune system in paraneoplastic pemphigus, a severe cancer-associated autoimmune disease that causes blisters on the skin and mucous membranes. To achieve this, we tested blood samples from the patients against thousands of human proteins to determine which ones were recognized by their antibodies. The findings provide new insights into how the immune system responds to cancer and can serve as early diagnostic markers to help detect cancer in patients with autoimmune blistering diseases.

## Abstract (web summary)

Eriksson, Aranda-Guillén et al., conduct a comprehensive analysis of autoantibodies in paraneoplastic pemphigus and identify that autoantibodies against SERPINB3 are associated with the life-threatening lung condition bronchiolitis obliterans. These discoveries provide new diagnostic biomarkers that could also allow for earlier detection of the underlying cancer.

## Introduction

Paraneoplastic syndromes encompass a wide range of conditions that develop secondary to cancer but are not explained by invasive growth of the tumor^1^. Instead, these syndromes are believed to develop primarily through the following mechanisms: 1) tumors may affect remote organs through the secretion of humoral factors, and 2) tumors may induce immune responses that cross-react with and injure healthy tissues^2,3^. Circulating autoantibodies are often detectable in paraneoplastic syndromes, pointing to an immune-mediated pathogenesis in these cases. Different mechanisms have, in turn, been proposed for the development of cancer-associated autoimmunity. The tumor may support the ectopic expression of tissue-specific proteins, where the immunogenic environment in the tumor triggers immune responses that also target the tissue with a native expression of these proteins. Ectopic expression of neuronal proteins has been demonstrated in tumors from patients with paraneoplastic neurological syndromes, supporting this notion^2^. Alternatively, in another scenario, tumors may express a mutated gene product that is recognized as foreign by the immune system, resulting in an immune response that cross-reacts with the corresponding native protein in a remote organ^4^.

The skin and mucous membranes are frequent targets of cancer-associated autoimmunity^5^. Paraneoplastic pemphigus is a severe autoimmune bullous disease that manifests with polymorphic lesions affecting both the skin and the mucous membranes^6^. The clinical presentation is variable and may resemble pemphigus vulgaris, lichen planus or erythema multiforme^7^. Painful mucositis typically affects all patients and is the initial symptom in almost half of the cases^8^. Alongside the mucocutaneous manifestations, a subset of patients develops bronchiolitis obliterans, which often leads to fatal respiratory failure^9,10^. Malignant lymphoma is the most common form of neoplastic disease found in this group of patients, though various other malignancies and benign tumors have also been reported^3^. Often, the underlying tumor remains undiagnosed at the time when the mucocutaneous manifestations appear. Prompt and accurate diagnosis is crucial to initiate investigations for occult malignancies and start appropriate treatment without delay. Autoantibody analysis constitutes a cornerstone of the diagnostic assessment of patients with autoimmune blistering diseases, including paraneoplastic pemphigus. Traditionally reliant on indirect immunofluorescence, this serological screening has expanded to include a growing range of antigen-specific tests^11^. We recently identified the epidermal protein transglutaminase 1 (TGM1) as a disease-specific autoantibody target in paraneoplastic pemphigus^12^. Despite these advances, the spectrum of autoimmune targets in paraneoplastic pemphigus has not been systematically explored. Particularly, the differences in autoantibody repertoires between patients with paraneoplastic versus non-paraneoplastic autoimmune blistering disease are not well understood. Moreover, the mechanisms behind the severe respiratory complications observed in many paraneoplastic pemphigus patients are still unclear. To address these gaps, we here used a systematic approach to proteome-wide autoantibody profiling and independent validation in patients with paraneoplastic pemphigus.

Here, we show that paraneoplastic pemphigus features a broad repertoire of disease-specific autoantibodies that mainly target the clinical manifestation sites, particularly in the skin and other squamous epithelia. The autoantibody spectrum is largely similar across different tumor types, except in thymoma, where patients present a distinct profile with autoantibodies against cytokines. Notably, we identify SERPINB3 as a major autoantibody target with an expression pattern and clinical association suggesting a role in bronchiolitis obliterans.

## Methods

### Clinical subjects

Patients diagnosed with paraneoplastic pemphigus (*n* = 84) at Kurume University Hospital were included in the study (Supplementary Table S8). The diagnosis was made based on the clinical information obtained through referrals and rigorous immunoserological diagnostic tests, including immunoblotting and indirect immunofluorescence of healthy skin tissue with patient serum, as previously described^13^. The diagnostic criteria for paraneoplastic pemphigus require the detection of antibodies against both envoplakin and periplakin. Additionally, indirect immunofluorescence using rat bladder tissue was performed, with ~70% of paraneoplastic pemphigus sera showing positivity. Representative images from direct and indirect immunofluorescence studies in a patient with PNP are shown in Supplementary Fig. S12. Some patients who met the immunodiagnostic criteria for paraneoplastic pemphigus did not have a known neoplasia at the time of diagnosis. Patients with pemphigus vulgaris (*n* = 19), bullous pemphigoid (*n* = 20), pemphigus foliaceous (*n* = 20), epidermolysis bullosa acquisita (*n* = 14) and Ap200P (*n* = 30) were included from the same biobank at Kurume University^14^. The diagnostic procedures and patient classifications for these groups were based on a combination of clinical documentation (e.g., consultation letters), indirect immunofluorescence on normal and NaCl-split human skin, immunoblotting with epidermal extracts and recombinant proteins, and commercial ELISA assays, as previously described^14^. Swedish blood donors and patients with pancreatic cancer with no known history of autoimmune disorders were included as controls.

### Microarray

Solid phase protein arrays featuring >9000 full-length human proteins (ProtoArray v5.1, PAH05251020, ThermoFisher, Waltham, Mass) were screened with diluted 1:2000 sera from patients affected by paraneoplastic pemphigus (*n* = 14), Ap200P (*n* = 8), and 1 healthy control. Formed immunocomplexes were detected with a secondary antibody fluorescent signal (Alexa Fluor 647 goat anti-human IgG, A21445, ThermoFisher) diluted 1:2000. Manufacturer’s protocol was followed using recommended reagents: ProtoArray Blocking Buffer Kit (PA055, ThermoFisher), Dylight 550 goat anti-GST (#DY550011-13-001), Cayman chemicals, Ann Arbor, Mich) diluted 1:10000. Microarrays were scanned using the LuxScan HT24 (BioCapital) scanner. Additional data from 17 healthy donors were included from previous microarray experiments conducted under the same experimental conditions.

Proteins were printed in duplicates on the microarray. The result for one sample was defined as the maximum of the duplicates:

$${{Singal}}_{{Sample}}=\max \left\{\begin{array}{c}\begin{array}{cc}{{Signal}}_{{Protein}} & {duplicate}1\end{array}\\ \begin{array}{cc}{{Signal}}_{{Protein}} & {duplicate}2\end{array}\end{array}\right.$$

### Bead-array

The top-scoring candidate autoantigens in the microarray were purchased in solution and used in a bead-based array to screen serum samples for autoantibodies (Supplementary Note I). Briefly, 3 μg of protein was coupled with 1,500,000 magnetic beads (MagPlex®, Luminex Corp.) using AMG Activation Kit for Multiplex Microspheres (AnteoTech, A-LMPAKMM). Patient serum samples were diluted 1:25 in PBS and, subsequently, 1:10 in 0.05% PBS-T, 3.3% BSA and 5.5% milk, to finally incubate them with coupled beads for 2 h at room temperature. An R-phycoerythrin-labeled goat anti-human IgG (Invitrogen, H10104) was used diluted 1:625 as a secondary antibody to detect autoantibody binding by measuring the median fluorescent intensity in a FlexMap 3D (Luminex Corp.).

### Radioligand binding assay

Autoantibody presence towards SERPINB4 was also studied by immunoprecipitation. Antigen’s cDNA was purchased from OriGene (SC321095) and subcloned into pSp64 vector (P1241, Promega). The plasmids were transcribed and translated in vitro using radiolabeled 35S-methionine (PerkinElmer). Incubation of 2.5 μl of patient serum in conical 96-well plates and 30,000 cpm of radiolabeled protein per well was performed overnight at 4 °C. The immunoprecipitation was prepared in 96-well filter plates (MultiScreen Merck Millipore, 1.2 μm hydrophilic PVDF) using protein A sepharose (4 Fast Flow, GE Healthcare) as a filter retainer of immunocomplexes during 45 minutes incubation at 4 °C. Microbeta counter (1450 Microbeta Triplex, Wallac) read the photon emission of scintillation fluid (PerkinElmer, 1200.437) where radioactive immunocomplexes were submerged. 4% BSA was used as a negative control, and a patient with paraneoplastic pemphigus positive for SERPINB4 autoantibodies in the microarray was used as a positive control. Studied patient samples were pipetted in duplicates.

### Statistics

For the microarray experiment, quantile normalization was used to control for technical variation among different arrays and experiments. The average signal intensity was calculated across cases and controls separately:

1 $${\mu }_{{SignalCases}}=\frac{1}{{n}_{{cases}}}\mathop{\sum }\limits_{i=1}^{{n}_{{Cases}}}{{Signal}}_{{Sample}}{in\; cases}$$

2 $${\mu }_{{SignalControls}}=\frac{1}{{n}_{{controls}}}\mathop{\sum }\limits_{i=1}^{{n}_{{Controls}}}{{Signal}}_{{Sample}}{in\; controls}$$

The fold change between cases and controls was defined as follows, where *μ* is the mean signal in cases and controls, as indicated.

3 $${{\log }_{2}({{\rm{Fold\; change}}})=\log }_{2}\frac{{\mu }_{{SignalCases}}}{{\mu }_{{SignalControls}}}$$

Proteins were ranked by log_2_ fold change.

In the bead-array assay, a cutoff consisting of either the mean +5 standard deviations for each antigen in healthy controls or at least 500 units MFI was established to define those samples considered positive for the presence of autoantibodies. The most informative autoantigens were selected based on the best performance at discriminating cases with PNP from other diseases and healthy controls, evaluated with the area under an ROC curve (AUC). Antigens with an AUC > 0.50 were analyzed in combinations in silico to create an optimal panel to separate cases and controls, based on the bead-array data. All possible combinations of antigens, from 1 antigen to 15, were obtained following the formula:

$$\frac{n!}{\left(n-r\right)!r!}$$

where *n* is the total number of antigens (*n* = 15), and *r* is the number of antigens to be combined. For instance, in combinations of 5, 3003 different combinations were analyzed. The combinations with the highest AUC were selected for presentation in Supplementary Table S2. Only one IFN type I and one IFNL2 were included to avoid inclusion of cross-reactive antigens.

The following expression was used to calculate antibody index in the radioligand binding assay: ((mean sample cpm–mean negative control cpm) ÷ (mean positive control cpm−mean negative control cpm)) × 100. A cut-off to determine the presence of autoantibodies was arbitrarily decided at three standard deviations above the mean of the blood donors' index.

### Study approval

All clinical subjects gave their informed consent. The study was approved by the Swedish Ethical Review Authority (Dnr 2019-03508, Dnr 2018/1792-31/2) and performed in accordance with the Declaration of Helsinki.

## Results

### Proteome-wide autoantibody repertoire analysis in patients with paraneoplastic pemphigus vs non-paraneoplastic blistering disease

**Fig. 1:**
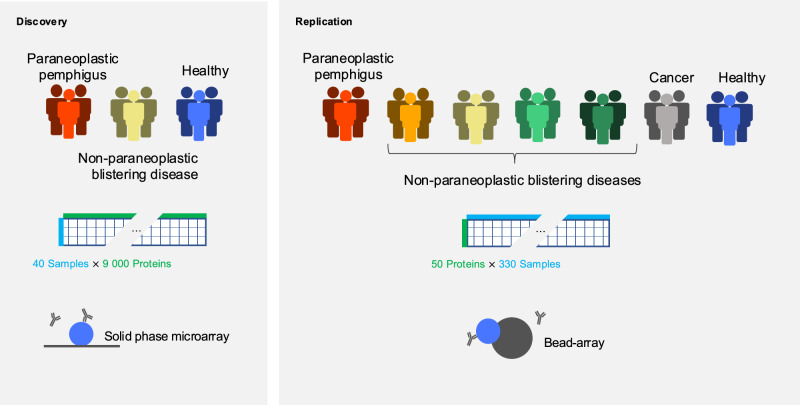
Autoantibody repertoire analysis and validation in paraneoplastic pemphigus. Characterization of the autoantibody repertoire in paraneoplastic pemphigus was conducted using a two-step approach. In the discovery phase, a cohort of 14 cases of paraneoplastic pemphigus, eight cases of Ap200P, and 18 healthy controls were screened with a microarray featuring more than 9000 full-length human recombinant proteins. In the second phase, the newly identified putative autoantigens were validated using a bead-based array in an extended cohort of paraneoplastic pemphigus patients, in patients with non-paraneoplastic blistering diseases, cancer, and in healthy controls.

**Fig. 2:**
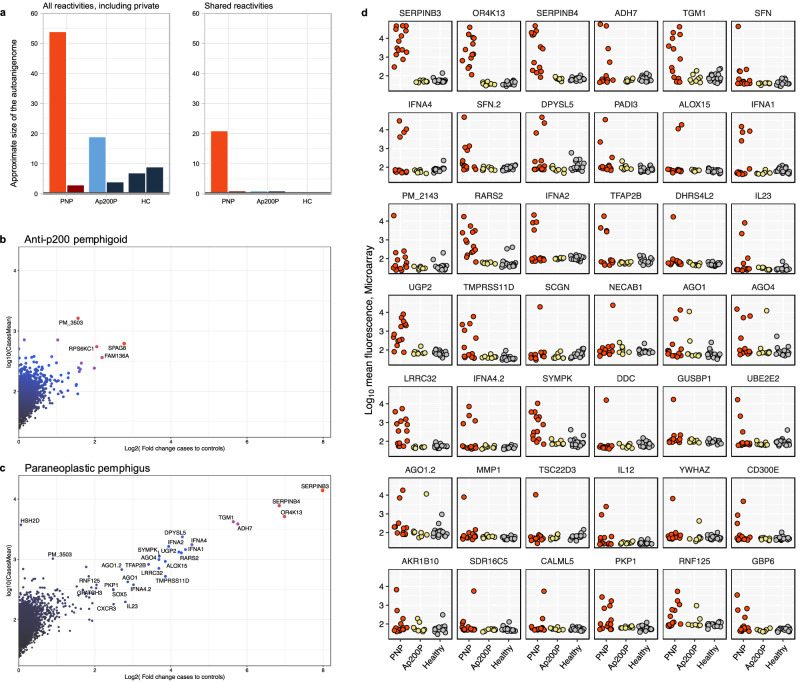
Paraneoplastic pemphigus features a broad repertoire of disease-specific autoantibodies. **a** The barplots display the approximated size of the autoantigen repertoire (*y* axis) in paraneoplastic pemphigus (PNP), anti-p200 pemphigoid (Ap200P), and healthy controls (left and right barplots). In comparison of PNP and healthy controls, proteins with stronger signals in PNP are shown in bright red, while those stronger in healthy controls are in dark red. Similarly, the comparison between Ap200P and healthy controls is shown in light blue, while internal comparisons between equal-sized subsets of healthy controls are shown in dark blue. Autoantibody signals exceeding a signal intensity of case-control enrichment (absolute log2 fold-change) of 2.5 or greater were considered positive. To identify autoantibody targets that were shared between two or more patients, we performed analyses leaving out the sample with the strongest signal from the calculation (right plot). **b**, **c** The panels display the top results from the microarray screening in the discovery dataset. Proteins at the top right displayed strong autoantibody signals in at least two patients with Ap200P (**b**) and paraneoplastic pemphigus (**c**), respectively. **d** displays the autoantibody fluorescence signal, and the 42 facets highlight the proteins with the largest case-control fold-change difference in paraneoplastic pemphigus compared with healthy controls.

We used a two-step approach to characterize the autoantibody repertoire in paraneoplastic pemphigus (Fig. 1). Serum samples from patients with paraneoplastic pemphigus (*n* = 84) and other forms of autoimmune blistering (*n* = 103) diseases were included from a previously described cohort in Japan^13,14^. Separately, we included patients with pancreatic cancer and no known paraneoplastic manifestations (*n* = 38) and blood donors (*n *= 105) as additional control groups. The paraneoplastic pemphigus patient cohort was divided into a discovery cohort consisting of 14 cases, subjected to proteome-wide autoantibody screening, and a replication cohort comprising 70 cases, to independently validate the identified autoantibody targets. We wanted to compare the autoantibody repertoires in paraneoplastic pemphigus to those of patients with autoimmune blistering diseases without neoplastic association. To this end, we included eight patients with the non-paraneoplastic autoimmune blistering disease anti-p200 pemphigoid (Ap200P) in the proteome-wide autoantibody analysis. These two groups were compared to a total of 18 healthy blood donors. Autoantibody screening was conducted using protein microarrays featuring over 9000 full-length human proteins. We assessed the fluorescence signal intensity and the case-control enrichment (fold change) to define the autoantibody repertoires in paraneoplastic pemphigus and Ap200P. Paraneoplastic pemphigus stood out with a much greater number of autoantibody reactivities compared to Ap200P, regardless of the cut-off used (Fig. 2a). At a log_2_ fold change of 2.5 (equating to five- to sixfold enrichment), paraneoplastic pemphigus displayed autoantibody reactivity to 54 proteins, as compared to 19 in Ap200P. These hits included both private reactivities (increased in one subject) and shared reactivities (increased in more than one subject). Since shared autoantibody reactivities are more likely to represent genuine disease-associated autoantibodies, we were interested to look further into the shared reactivities^15^. To estimate the autoantibody repertoires focusing on shared reactivities only, we repeatedly conducted leave-one-out analyses (Supplementary Figs. S1 and S2). For each protein, the sample with the strongest signal was excluded from case-control enrichment calculations. This revealed a striking separation of the disease groups, where paraneoplastic pemphigus displayed 21 candidate autoantigens that were shared between two or more patients, while Ap200P had only 1, and healthy controls had none. When we looked further into the hits shared between four patients and five patients, autoantibody reactivities were exclusively found in the paraneoplastic pemphigus group. In all, the proteome-wide autoantibody data suggested that paraneoplastic pemphigus featured a broad repertoire of disease-associated autoantibodies that greatly exceeded that observed for the non-paraneoplastic blistering disease Ap200P.

### Shared patterns of tissue expression and function among candidate autoantigens

In the ranked list of autoantibody targets in paraneoplastic pemphigus, there were many proteins showing highly increased reactivities in this patient group compared to controls (Fig. 2b, c). Two of the top hits were SERPINB3 and SERPINB4, both belonging to the serine protease inhibitor family. We reviewed the results for all serpins in the microarray (Serpins A3, A6, A10, A12, B2, B3, B4, B6, B7, B9, and E2) and could conclude that the autoantibodies were highly specific for B3 and B4 (Supplementary Fig. S4). Autoantibodies to SERPINB3 and SERPINB4 were found in close correlation (*R*^2^ 0.75, calculated on log-transformed signal intensities), with SERPINB3 autoantibodies showing the highest prevalence, as detected in 93% (13/14) of the patients with paraneoplastic pemphigus (Fig. 2d).

Plakophilin-1 (PKP1) and the plakophilin interacting protein stratifin (SFN), were identified as new putative autoantigens in paraneoplastic pemphigus. Interestingly, these two proteins are known to interact and are important for epithelial barrier integrity^16,17^. SFN exhibited consistent results across two isoforms present on the microarray. The recently described autoantigen TGM1 was confirmed^12^. Additionally, cytokine autoantibodies targeting type I interferons (IFNA4, IFNA1, IFNA2), and interleukins 23 and 12 were detected in 4 out of 14 patients in the discovery cohort (Fig. 2d, Supplementary Fig. S5). The exploratory autoantibody screening thus confirmed known autoantibodies associated with paraneoplastic pemphigus and also identified novel disease-specific autoantigen candidates (Supplementary Note II). However, due to the finite coverage of the protein arrays, some established autoantigens, such as envoplakin and periplakin, could not be assessed.

**Fig. 3:**
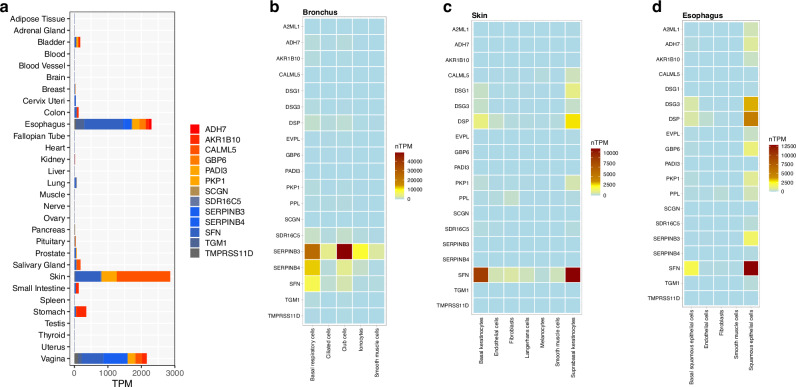
Tissue expression patterns of autoantibody target genes. **a** Gene expression levels (Transcripts Per Million, TPM) of major autoantibody targets identified in paraneoplastic pemphigus, showing a marked enrichment in the skin and other squamous epithelia (data from the GTEx database, v6p.v1.1.8). Each bar comprises stacked boxes, with the width of each box representing the interquartile range of gene expression in the corresponding tissue. **b**–**d** Heatmaps showing normalized single-cell transcript expression levels (nTPM), summarized per gene and cell type cluster for selected tissues, highlighting SERPINB3 expression in bronchiolar cells. Single-cell expression data were obtained from the Human Protein Atlas.

Several of the top hits were known to play a role in desmosome function and in maintaining the integrity of the epithelial barrier. To understand the functional relationships between the autoantibody targets, we assessed their tissue distributions using RNA-sequencing data from the GTEx portal. This revealed a shared expression pattern among the top-scoring autoantigens, primarily localizing to the epithelia of the skin, esophagus, and vagina (Fig. 3a, Supplementary Fig. S6). Alpha interferons (IFNA1, IFNA2, IFNA4) showed no expression in the investigated tissues, while interleukins 12 and 23 displayed modest expression in lymphocytes. We next leveraged single-cell data from the Human Protein Atlas for a more detailed investigation of expression patterns^18^. Interestingly, SERPINB3 and SERPINB4 exhibited robust expression in bronchial tissues compared to other paraneoplastic pemphigus autoantigens, which are predominantly expressed in squamous epithelial cells of the esophageal mucosa or dermal keratinocytes (Fig. 3b–d). These findings align with the multisite manifestations seen in paraneoplastic pemphigus, which involve the skin, mucous membranes, and bronchi. Besides SERPINB3 and SERPINB4, no other autoantigen exhibited its highest expression in the bronchus, suggesting a potential link between SERPINB3/4 autoantibodies and bronchiolitis obliterans.

### Validation of autoantibodies in an extended cohort of autoimmune blistering diseases

To confirm the identified candidate autoantigens, we conducted an independent validation using a bead-based autoantibody assay in an extended group of patients with autoimmune blistering diseases. The validation cohort included patients with paraneoplastic pemphigus (*n* = 84, including the initial 14 from the discovery cohort), Ap200P (*n* = 30, including 8 from the discovery cohort), and healthy controls (*n* = 105, including 18 from the discovery cohort). To assess the specificity of the identified autoantibodies among clinically similar non-paraneoplastic autoimmune blistering skin diseases, we included patients with pemphigus vulgaris (PV, *n* = 19), pemphigus foliaceus (PF, *n* = 20), bullous pemphigoid (BP, *n* = 20), and epidermolysis bullosa acquisita (EBA, *n* = 14). In addition, we included patients with cancer but without apparent autoimmune manifestations (CA, *n* = 38).

**Fig. 4:**
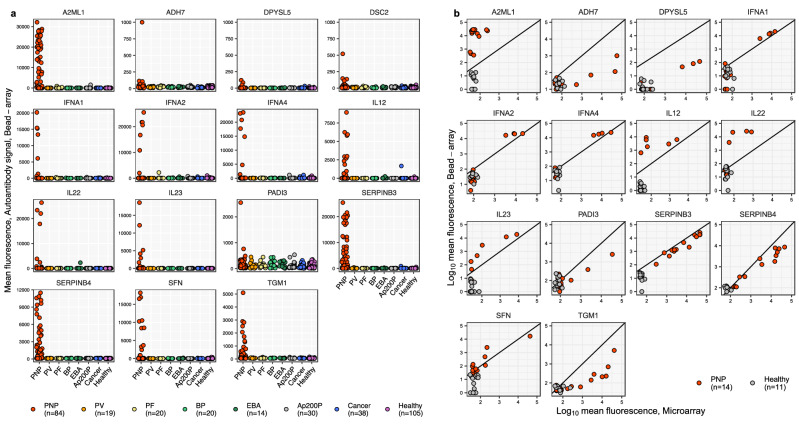
Validation of autoantibodies across different blistering diseases and cancer. **a** The identified autoantibodies were validated using a bead-based assay in an extended cohort. The replication analysis included patients with paraneoplastic pemphigus (PNP, *n *= 84), pemphigus vulgaris (PV, *n *= 19), pemphigus foliaceus (PF, *n* = 20), bullous pemphigoid (BP, *n* = 20), epidermolysis bullosa acquisita (EBA, *n* = 14), anti-p200 pemphigoid (Ap200P, *n* = 30), cancer (CA, *n* = 38), and in healthy controls (HC, *n* = 105). **b** Correlation of autoantibody signal intensities detected by solid phase microarrays (*x* axis) and bead-arrays (*y* axis) in patients with paraneoplastic pemphigus (PNP, *n* = 14) and healthy controls (*n* = 11). Facets display results for autoantigens selected for follow-up studies, ordered alphabetically.

In this extended cohort, we could successfully replicate several of the newly identified autoantibodies (Fig. 4a), with consistent results between the initial screening and validation (Fig. 4b). Autoantibodies against SERPINB3 were detected in 53% of paraneoplastic pemphigus patients, A2ML1 in 53%, SERPINB4 in 40%, SFN in 15% and TGM1 in 17%, each with a false discovery rate of 0% (*n* = 84). Cytokine autoantibodies were detected in 8–10% of the patients with paraneoplastic pemphigus. Autoantibodies to SERPINB4 were also confirmed by a radio-ligand binding assay using immunoprecipitation as the standard method used to test for antibodies against known paraneoplastic pemphigus antigens (Supplementary Fig. S7). The autoantigens PADI3 and ADH7 were replicated in the validation analysis, with consistent results across methods. However, no new cases of PADI3 and ADH7 autoantibody positivity were identified in the replication cohort, indicating that these autoantibodies are infrequent in paraneoplastic pemphigus (Supplementary Table S1).

Having identified several new disease-associated autoantibodies, we sought to determine whether these markers could provide a high diagnostic sensitivity and specificity for paraneoplastic pemphigus among clinically similar conditions. To this end, we combined bead-array data for all possible sets of antigens, aiming to identify the most informative autoantigen panel for distinguishing patients with paraneoplastic pemphigus from non-paraneoplastic blistering diseases and healthy controls. A positive detection of autoantibodies against any of the antigens in the panel was considered indicative of paraneoplastic pemphigus. We observed that the sensitivity improved with the inclusion of more autoantigens in the panels, stabilizing at about 76% (Supplementary Table S2). Given the high specificity of all autoantigens, the optimal panel achieved a specificity of 98% (Supplementary Table S3). In comparison, the clinically established diagnostic markers desmoglein 1 and desmoglein 3 autoantibodies showed a sensitivity of 64% for diagnosing paraneoplastic pemphigus. Incorporating the panel of new autoantigens increased this sensitivity to 89% (Supplementary Fig. S8).

### Paraneoplastic pemphigus autoantigen gene expression and mutations in tumors

A variety of malignancies and benign tumors have been reported in patients with paraneoplastic pemphigus. In our cohort, patients with paraneoplastic pemphigus were diagnosed with the following malignancies or benign tumors: 39 cases of lymphoma, 13 with solid tumors, 4 with thymoma, and 1 with leukemia. The specific cancer type was not determined in 27 cases. Among the diagnosed lymphomas, 11 were classified as follicular lymphoma, 18 as unspecified lymphoma, 4 as diffuse large B-cell lymphoma, and 6 as Castleman disease^13,14^.

**Fig. 5:**
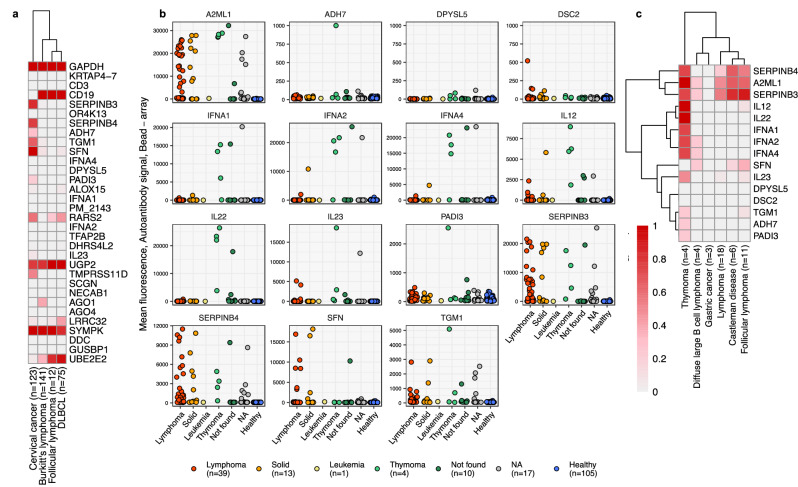
Expression and autoantibody profiles of paraneoplastic pemphigus autoantigens in lymphoma and other tumors. **a** Expression of paraneoplastic pemphigus (PNP) autoantigens in diffuse large B-cell lymphoma (DLBCL), follicular lymphoma, Burkitt’s lymphoma, and HIV-positive cervical cancer. The heatmap displays the proportion of samples with expression levels exceeding 10 RPKM. In addition to PNP autoantigens, the heatmap includes reference genes such as GAPDH (housekeeping gene), KRTAP4-7 (skin-specific), CD3 epsilon (T-cell-specific), and CD19 (B-cell-specific) for comparison. Tumor RNA-sequencing summary statistics were obtained from the NIH Center for Cancer Genomics and the Cancer Genome Characterization Initiative (CGCI), while RNA-seq data for normal tissues (ectocervix, endocervix, and EBV-transformed lymphocytes) were retrieved from the GTEx portal (v6p.v1.1.8). **b** Autoantibody signal intensities in patients with paraneoplastic pemphigus, stratified by the type of neoplasm. Autoantibodies were measured using a bead-based protein array in patients with paraneoplastic pemphigus (PNP, *n* = 84) and healthy controls (HC, *n* = 105). **c** The heatmap displays the proportion of samples with an autoantibody fluorescence signal >1000, categorized by the type of neoplasm group in patients with paraneoplastic pemphigus (*n* = 46). Only neoplasms present in two or more patients are included. The color scale is consistent across heatmaps (**a**) and (**c**).

One of the main hypotheses for the development of paraneoplastic autoimmunity proposes that the ectopic expression of tissue-specific proteins within tumors can induce autoimmunity targeted at organs where these proteins are normally expressed. To evaluate this hypothesis for paraneoplastic pemphigus, we investigated the expression of paraneoplastic pemphigus autoantigens in hematological tumors, leveraging transcriptomic sequencing from non-Hodgkin lymphomas through the Cancer Genome Characterization Initiative (CGCI) Data Matrix (Center for Cancer Genomics, NIH). We also included cervical cancer, expected to express epithelial proteins implicated in paraneoplastic pemphigus. For comparison, we used positive controls (GAPDH and CD19) and negative controls (KRTAP4-7 and CD3) (Fig. 5a). As expected, lymphomas consistently expressed CD19 but did not express CD3 or KRTAP4-7, whereas cervical cancer expressed typical ectocervical epithelial proteins. Overall, the protein expression profiles aligned with those of corresponding healthy tissues (lymphocytes and cervix), and there was no evidence of ectopic expression of paraneoplastic pemphigus autoantigens in tumors. SERPINB3 and SERPINB4 were expressed in most cervical cancers, in line with their native expression in squamous epithelia.

We next assessed the expression of the candidate autoantigen genes across various solid tumors, utilizing the molecular pathology data from the Human Protein Atlas (version 23.0)^18^ (Supplementary Fig. S9). Concordant with the CGCI transcriptome data, cervical cancers showed a high expression of SERPINB3 and SERPINB4. In addition, the expression of these genes in pancreatic cancer was associated with an unfavorable prognosis, reflecting SERPINB3’s role in promoting disease progression by inducing a basal-like/squamous subtype^19^.

Another proposed mechanism for paraneoplastic autoimmunity posits that tumor neoantigens can trigger immune responses that inadvertently target the corresponding native proteins in healthy tissues. Earlier findings that SERPINB3 is frequently mutated in cancers lend support for this hypothesis^20,21^. To explore this more systematically, we utilized the Tumor-specific Neoantigen Database (2023) to compare the number of neoantigen calls for paraneoplastic pemphigus autoantigens with those from non-paraneoplastic autoimmune skin diseases like pemphigus vulgaris/foliaceus or bullous pemphigoid^22^. Our analysis found no significant differences in the number of neoantigens between these groups and no overall enrichment of paraneoplastic pemphigus autoantigens within the neoantigen database (p-value 0.09 (Wilcoxon rank sum test), Supplementary Fig. S10a, b).

### Autoantibody profiles in relation to cancer type in paraneoplastic pemphigus

We stratified patients by their primary neoplasm and investigated autoantibody signal intensity using bead-based arrays in paraneoplastic pemphigus (*n* = 84) and healthy controls (*n* = 105) (Fig. 5b). Autoantibodies against SERPINB3 and SERPINB4, as well as A2ML1, were detected in patients with paraneoplastic pemphigus across all categories of neoplasms, including 39 lymphomas, 13 solid tumors, and 4 thymomas, except for a single patient with leukemia. Thymoma patients exhibited reactivity against cytokines, including type I IFN, IL12, IL22, and IL23, which were largely absent in the rest of the cohort. Additionally, we identified one patient with no available information regarding the type of underlying neoplasia whose autoantibody profile, reactive to multiple cytokines, was suggestive of thymoma.

We further stratified the patients with lymphomas into subtypes: Castleman (*n* = 6), follicular lymphoma (*n* = 11), diffuse large B-cell lymphoma (*n* = 4), and unspecified lymphoma (*n* = 18) (Fig. 5c). Interestingly, patients across all lymphoma types and thymoma showed reactivity to SERPINB3, SERPINB4 and A2ML1, whereas cytokine autoantibodies were detected almost exclusively in thymoma patients.

**Fig. 6:**
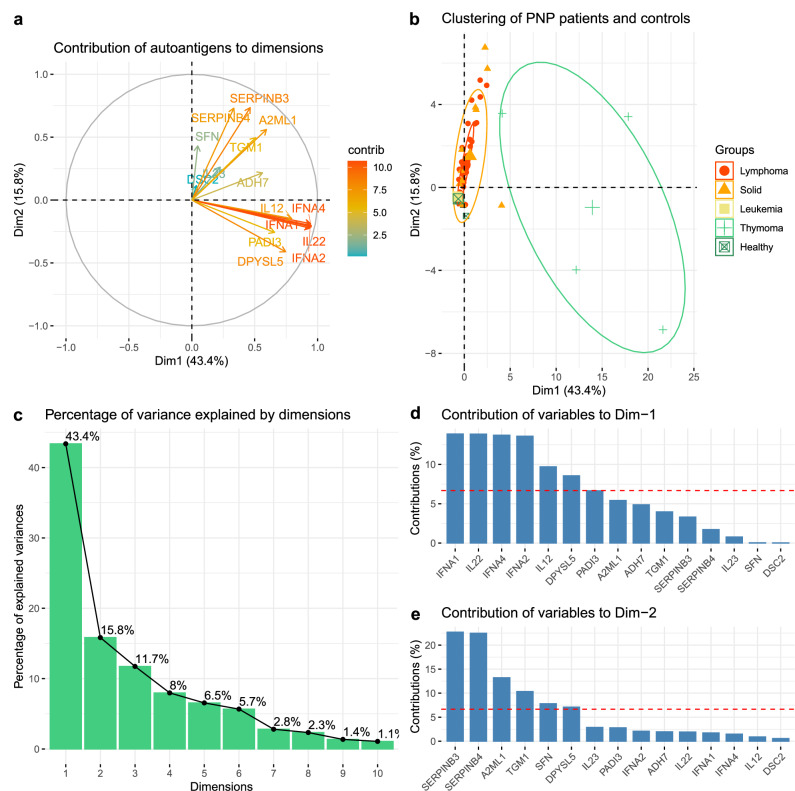
Principal component analysis of autoantibody profiles across tumor types in paraneoplastic pemphigus. **a** Arrows indicate the direction and magnitude of the most significant autoantibody contributions (contrib) in the PCA space. **b** Clusters of subjects with similar results. Patients with paraneoplastic pemphigus secondary to thymoma exhibit distinct autoantibody profiles, characterized by cytokine autoantibodies influencing dimension 1. **c** Scree plot showing the variance explained by each principal component. **d**, **e** Autoantigens contributing to the main axes.

Principal component analysis of the autoantibody signal intensities revealed two major profiles in autoantibody reactivity. First, it confirmed the distinctive profile of thymoma patients, driven largely by their reactivity to IFNA1, IL22, IFNA4, and IFNA2 (Fig. 6a, b, d). A second significant dimension separated patients with autoantibodies against SERPINB3 and SERPINB4, highlighting the correlation between these two autoantigens (Fig. 6a, e). Together, the presence of cytokine and SERPINB3/4 autoantibodies explained 59% of the dataset’s variability related to the underlying malignancies (Fig. 6c). The third and fourth components captured antigen signals too rare to be associated with specific tumor subtypes (Supplementary Fig. S11a–e).

### Bronchiolitis obliterans is associated with autoantibodies to SERPINB3

Bronchiolitis obliterans is a feared complication of paraneoplastic pemphigus with significant mortality. Autoantigens identified thus far in paraneoplastic pemphigus have exhibited expression patterns linking them with epithelial blistering, but not with bronchiolitis. Given the expression of SERPINB3 in bronchiolar cells, we investigated whether SERPINB3 autoantibodies were associated with the development of bronchiolitis obliterans in paraneoplastic pemphigus. We performed association analyses to explore the relationships between the autoantibody profiles, the type of neoplasms, and the expression of bronchiolitis obliterans, with a focus on SERPINB3 and IFNA1 autoantibodies, as they accounted for most of the variance in autoantibody profiles. Notably, the strongest association involving SERPINB3 was observed with bronchiolitis obliterans (p-value 0.009 (Fisher's exact test), Supplementary Table S4), with SERPINB3 autoantibodies detected in 14 (82%) of the 17 patients with bronchiolitis obliterans. Among the neoplasms, follicular lymphoma exhibited the strongest association with SERPINB3 autoantibodies (p value 0.02, Fisher's exact test). An association between autoantibodies against A2ML1 and bronchiolitis obliterans was also found (*p* value 0.003, Fisher exact test, Supplementary Table S5), while the same trend was observed with SERPINB4 (Supplementary Table S6).

Autoantibodies to IFNA1 were detected in three (75%) of the four patients with confirmed thymoma, and in another patient with putative thymoma, whereas the remaining patients were negative for these autoantibodies (Supplementary Table S7). A correlation between thymoma and antibodies against IL22, IL23 and IL12 was also found (*p* value 9e-05, 0.003, 4e-04, respectively (Fisher's exact test)).

## Discussion

The significance of anti-tumor immunity in cancer defense has been debated, but the transformative success of checkpoint inhibitors in cancer treatment has established its crucial role in the natural defense against malignancies. However, these immune responses are sometimes misdirected toward healthy tissues, leading to severe autoimmune paraneoplastic syndromes in patients with cancer. Understanding the immunological mechanisms underlying paraneoplastic autoimmunity is important for the diagnosis and effective management of these disorders and can also provide insights into tumor immunology. This study employed a systematic approach involving proteome-wide screening and independent validation to characterize the autoantibody repertoire in paraneoplastic pemphigus compared to non-cancer-associated autoimmune blistering diseases. Several key conclusions can be drawn from our findings. First, paraneoplastic pemphigus stood out from non-cancer-associated blistering diseases with a much broader spectrum of disease-associated autoantibodies. The breadth of the autoantibody repertoire was comparable to that seen in severe monogenic autoimmunity syndromes, such as autoimmune polyendocrine syndrome type I (APS1)^23^ and Immunodysregulation polyendocrinopathy enteropathy X-linked syndrome (IPEX)^24^, pointing to a major breakdown in immune tolerance in paraneoplastic pemphigus. Secondly, there was a striking pattern for the identified autoantibody targets and their predominant expression in the clinical manifestation sites, particularly in the skin and other squamous epithelia. Several autoantibody targets were also functionally related, such as plakophilin-1 (PKP1) and stratifin (SFN), both playing roles in desmosomal function within the skin and mucous membranes^25^. A third key observation is that the autoantibody repertoires were largely similar across the different tumor types, suggesting that the autoimmune responses are primarily linked to the blistering disease manifestations rather than the underlying immune events within the tumors. However, thymoma stood out with a distinctive autoantibody profile, characterized by the presence of multiple cytokine autoantibodies, including autoantibodies against type I IFNs, IL22, IL12, and IL23.

The detection of cytokine autoantibodies in thymoma patients aligns with earlier studies^26^, and draws intriguing parallels to other disorders involving thymic defects and cytokine autoantibody production^26–31^. Emerging evidence underscores how such autoantibodies can profoundly impact immune responses and contribute to infectious disease susceptibility. For example, autoantibodies to type I IFNs have been linked to severe outcomes in viral diseases, while anti-IL23 antibodies are associated with persistent opportunistic infections, including mycobacterial, bacterial, and fungal diseases^32^. Unfortunately, we did not have information about infectious disease histories for the studied patients. Investigating potential infectious disease risks in patients with paraneoplastic pemphigus, especially in those with concomitant thymoma, should be a priority for future research.

While the majority of patients with paraneoplastic pemphigus have disease confined to the skin and mucous membranes, a subset develops the severe, life-threatening pulmonary complication bronchiolitis obliterans. This condition is often resistant to treatment and carries a poor prognosis, with limited therapeutic options beyond immunosuppression. The mechanisms underlying bronchiolitis obliterans in paraneoplastic pemphigus are poorly understood but are thought to involve immune-mediated destruction of the bronchiolar epithelium, with inflammation, fibrosis, and eventual obliteration of small airways^33^. To date, autoantigens in paraneoplastic pemphigus have been primarily associated with the skin and other squamous epithelia, and a known squamous epithelial autoantigen has been associated with bronchiolitis obliterans^34^. Our study identifies two major autoantibody targets, SERPINB3 and SERPINB4, which exhibit distinctive expression in bronchiolar epithelial cells, linking paraneoplastic pemphigus with airway-specific autoimmunity. Autoantibodies to SERPINB3 and SERPINB4 were found in association with bronchiolitis obliterans in our cohort, further supporting this link. However, while the presence of these autoantibodies is striking, our observational study cannot determine their functional role in bronchiolitis obliterans and future studies are needed to understand their potential pathological mechanisms.

Paraneoplastic mucocutaneous manifestations often present in individuals whose cancers have not yet been diagnosed^35^. Although paraneoplastic pemphigus has characteristic clinical features, typically manifesting with erosive mucositis that can be either isolated or seen in association with dermal eruptions, these cases are easily mistaken for the more common non-cancer-associated forms of autoimmune blistering diseases. This highlights the need for reliable biomarkers of paraneoplastic pemphigus to ensure timely investigations for occult malignancies and appropriate treatment. Our data, incorporating both newly identified and previously known autoantibodies, show a potential to improve diagnostic sensitivity and specificity in identifying paraneoplastic pemphigus cases among clinically similar blistering diseases, with a collective diagnostic sensitivity of 76% and a false discovery rate of 6%. However, further studies are needed to fully evaluate the added diagnostic value of integrating these novel biomarkers and biomarker panels into the assessment of patients with blistering diseases.

In contrast to autoantibodies targeting desmogleins that are known to exert direct pathogenic effects by binding extracellularly exposed epitopes^36^, it is unlikely that autoantibodies directed against intracellular proteins such as SERPINB3 and SERPINB4 are themselves pathogenic. Rather, the presence of SERPINB3/4 autoantibodies likely reflects an underlying cell-mediated immune response in which cytotoxic T cells recognize and eliminate epithelial cells expressing these intracellular proteins, as seen for other autoimmune diseases^37^. As such, these autoantibodies may serve primarily as biomarkers of T-cell–driven autoimmunity targeting squamous epithelia, rather than acting as direct mediators of tissue damage.

One of the leading hypotheses for the etiology of paraneoplastic autoimmunity suggests that immune responses to tumor neoantigens drive autoimmunity against the corresponding native proteins in healthy tissues. Interestingly, SERPINB3 and SERPINB4 have been found to be recurrently mutated in cancer^21^, lending support for this proposed mechanism. Along the same line, tumor mutations in the SERPINB3 and SERPINB4 genes have been associated with beneficial responses to checkpoint inhibitor therapy^21^. Similar observations have been made in paraneoplastic systemic sclerosis, where tumor mutations in genes encoding autoantibody targets have been identified^4^. In our investigation using public tumor databases, we were unable to find significant enrichment of paraneoplastic autoantigens among tumor neoantigens. Another hypothesis posits that tumors may support ectopic expression of tissue-specific proteins, which could trigger autoimmunity against the native tissues expressing those proteins. Observations in support of this have been made in patients with paraneoplastic neurological diseases^2^. In our study using public data on tumor gene expression, we did not find compelling evidence of paraneoplastic pemphigus autoantigens being expressed in associated neoplasms. However, an important limitation of our studies on neoantigens and ectopic gene expression was that we did not have access to tumor specimens from our patients and instead had to rely on published data from other patient cohorts. Future studies that directly compare autoantibody repertoires with tumor antigen expression and tumor mutations in the same patients will be important in further elucidating the mechanisms of paraneoplastic autoimmunity.

## Conclusions

In this study, we characterized the repertoire of immune targets in paraneoplastic pemphigus, revealing a tissue-specific pattern of autoantibody reactivity involving proteins expressed in the skin and mucous membranes. Importantly, we identified SERPINB3 as a major target of autoimmunity with an expression pattern and association linking it to the severe and poorly understood complication bronchiolitis obliterans. The overall autoantibody profiles were largely consistent across different tumor types, with the exception of thymoma, where patients displayed a distinct antibody signature involving cytokine autoantibodies. The identified autoantibodies were highly specific for paraneoplastic pemphigus compared with clinically similar non-paraneoplastic autoimmune blistering diseases and hold strong potential for early cancer detection in patients presenting with blistering disease.

## Supplementary information

Supplementary Information

Description of Additional Supplementary Files

Supplementary Data 1

## Data Availability

The data that support the findings of this study are available through the ArrayExpress collection in BioStudies under accession number E-MTAB-14920. Source data for all Figures can be accessed from Supplementary Data 1. RNA-seq expression data for healthy subjects were retrieved from the Human Protein Atlas^18^ version 23.0 (https://www.proteinatlas.org/), accessed 2023-12-13 (rna_single_cell_type.tsv, and rna_single_cell_cluster_description.tsv), and the GTEx portal (v6p.v1.1.8), accessed 2022-12-05. The GTEx database was queried using the top 42 autoantibody targets identified in Fig. 2d (Supplementary Fig. S6). From these, tissue-specific proteins were selected for further emphasis in Fig. 3a. RNA-seq summary statistics for hematological malignancies were retrieved from the CGCI Data Matrix (https://ocg.cancer.gov/programs/cgci/data-matrix), accessed 2022-12-09 (datasets BLGSP, HTMCP-CC, NHL-DLBCL, and NHL-FL). Frequent neoantigens were retrieved from the Tumor-specific Neoantigen Database^22^, version 2, accessed October 2023.
