# Supplementary material for: Autoantibody repertoire analysis in paraneoplastic pemphigus reveals novel targets linked to mucocutaneous blistering and bronchiolitis obliterans
Source: Commun Med (Lond). 2026 Jan 10;6:74. doi: 10.1038/s43856-025-01335-2 (PMC12873347; doi:10.1038/s43856-025-01335-2)
Supplement: Supplementary file 1 — Supplementary Information [file 43856_2025_1335_MOESM1_ESM.pdf]

SUPPLEMENTARY INFORMATION FOR:

Autoantibody repertoire analysis in paraneoplastic pemphigus reveals novel targets linked to mucocutaneous blistering and bronchiolitis obliterans

Table of Contents

Figure S1 – Autoantibody signal distributions across samples 3

Figure S2 – Estimated size of the autoantigen repertoire in PNP 4

Figure S3 – Negative control analysis of autoantibody signals 5

Figure S4 – Autoantibody specificity across serpin proteins 6

Figure S5 – Microarray signal intensities for top PNP-enriched proteins 7

Figure S6 – Tissue expression of putative autoantigens (GTEx) 8

Figure S7 – Validation of SERPINB4 autoantibodies by radioligand binding assay 9

Figure S8 – PNP sensitivity: Desmogleins alone vs. combined biomarkers 10

Figure S9 – Tumor expression and prognostic value of PNP autoantigens (HPA) 11

Figure S10 – Tumor neoantigen load for PNP and non-PNP autoantigens 12

Figure S11 – PCA of autoantibody profiles (Dimensions 3 & 4) 14

Figure S12 – Immunofluorescence for PNP diagnosis 15

Supplementary Table S1 – Autoantibody profile in patients with PNP (n = 84) 16

Supplementary Table S2– Optimal antigen sets for discriminating PNP cases 17

Supplementary Table S3 – Diagnostic performance of multi-antigen autoantibody panel 18

Supplementary Table S4 – Association of SERPINB3 autoantibodies to clinical disease manifestations 19

Supplementary Table S5 – Association of A2ML1 autoantibodies to clinical disease manifestations 19

Supplementary Table S6 – Association of SERPINB4 autoantibodies to clinical disease manifestations 20

Supplementary Table S7 – Association of IFNA1 autoantibodies to clinical disease manifestations *20*

Supplementary Table S8 – Summary of clinical features of the PNP cohort (n = 84) *21*

Supplementary Note I – Recombinant proteins included in the bead-array experiment *23*

Supplementary Note II – List of PNP autoantigens identified with protein microarrays *24*

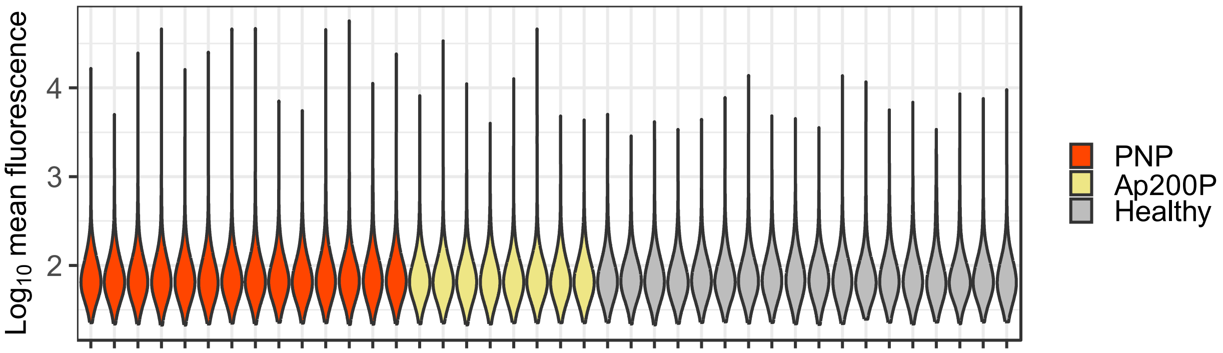

Figure S1 – Autoantibody signal distributions across samples

Distribution of mean fluorescence signal intensities for 9,312 proteins from 7,655 unique genes in the discovery data set. Violin plots are coloured by sample group; patients with paraneoplastic pemphigus (PNP, n=14), Anti-laminin gamma1 pemphigoid (Ap200P, n=8), and healthy controls (n=18).

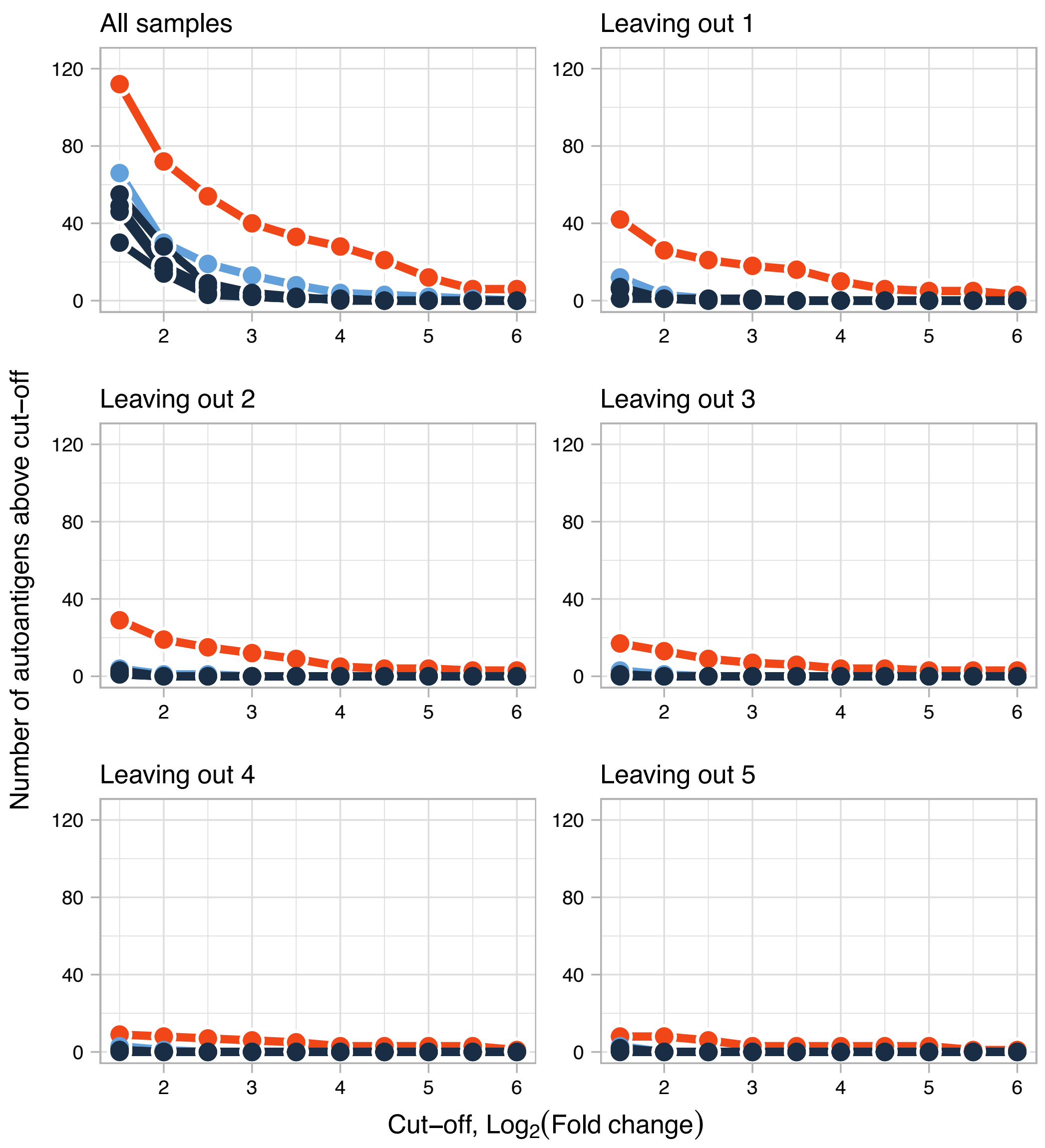

Figure S2 – Estimated size of the autoantigen repertoire in PNP

The panel displays the approximate size of the autoantigen repertoire in paraneoplastic pemphigus (y-axis, red line/points). The x-axis shows the cut-off for fold change, i.e. the level of enrichment in cases required to define a potential candidate autoantigen. The comparison of PNP vs healthy controls are shown in red: proteins enriched in PNP are shown in bright red, and stronger in healthy controls in dark red. The comparison of Ap200P vs healthy controls are shown in light blue. Internal comparisons between equal sized subsets of healthy controls are shown in dark blue. Facets display the dataset once 0-5 samples with the strongest signal for each protein has been left out of the calculations. Hence, facets 2-6 investigate the repertoire of shared reactivities.

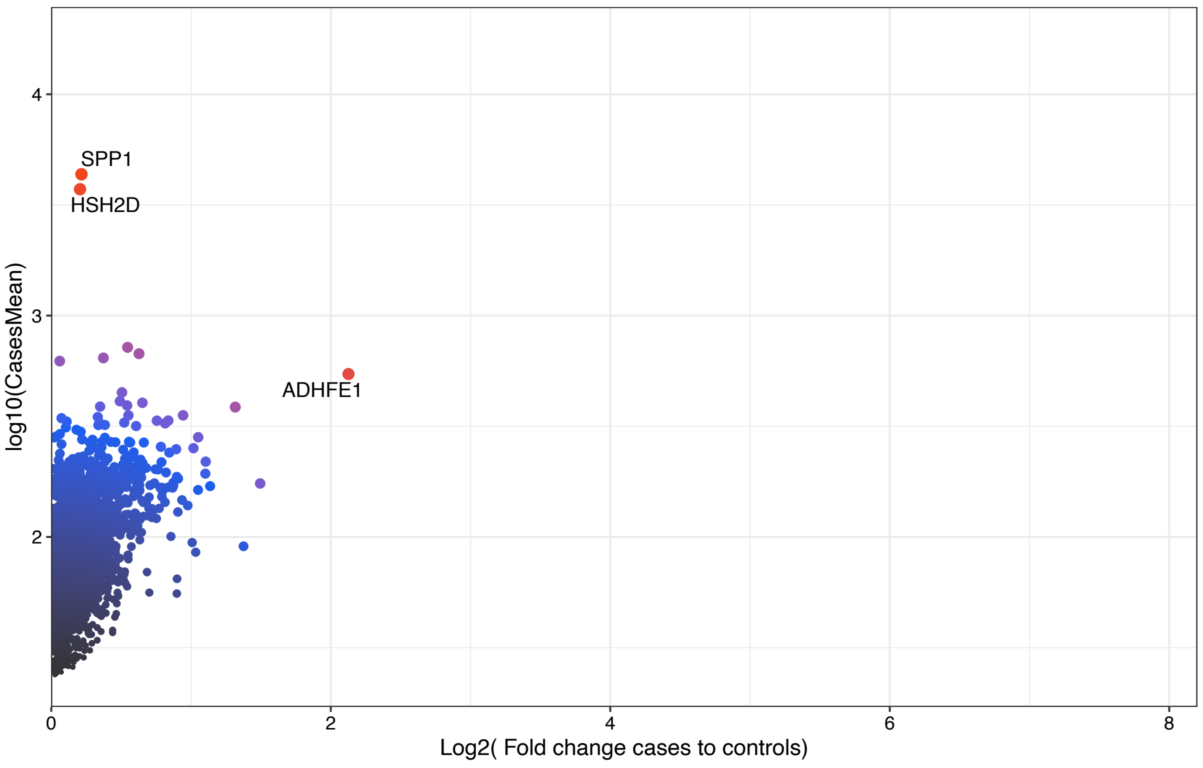

Figure S3 – Negative control analysis of autoantibody signals

The figure shows the top results from a negative control analysis using microarray data from the discovery cohort. In this analysis, the healthy control group was randomly split into two halves and compared against each other. Proteins with strong autoantibody signals in at least two individuals would appear in the top right corner. This approach allows us to assess background variability in groups where no enrichment of pathological autoantibodies is expected.

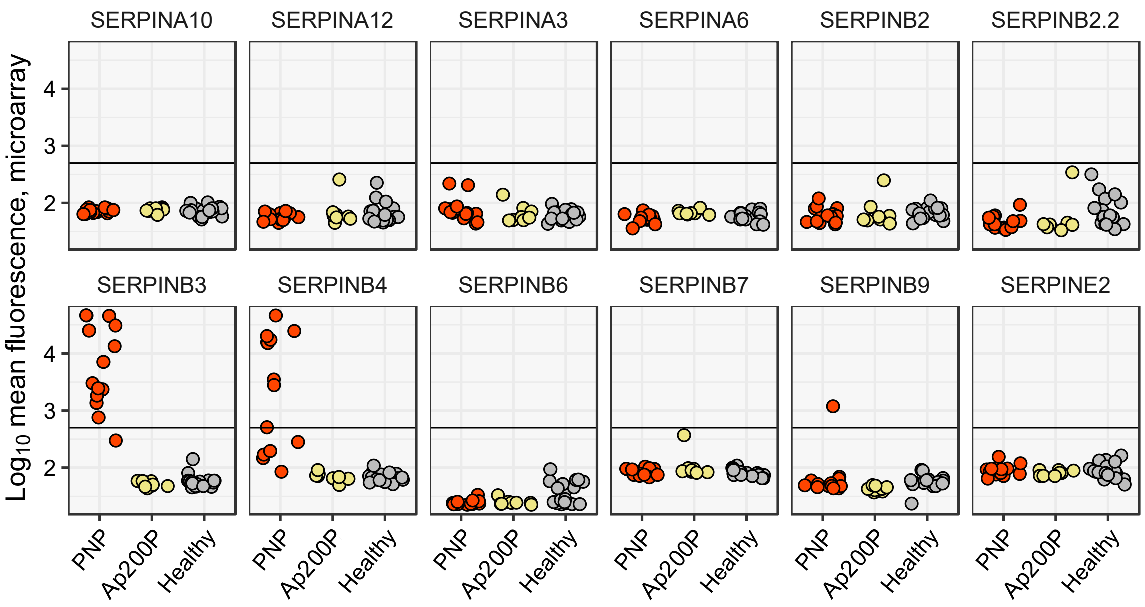

Figure S4 – Autoantibody specificity across serpin proteins

Specificity of autoantibodies across serpin proteins on the microarray: n = 14 (PNP), 8 (Ap200P), 18 (Healthy). The horizontal black line is set at $\log_{10}(500)$.

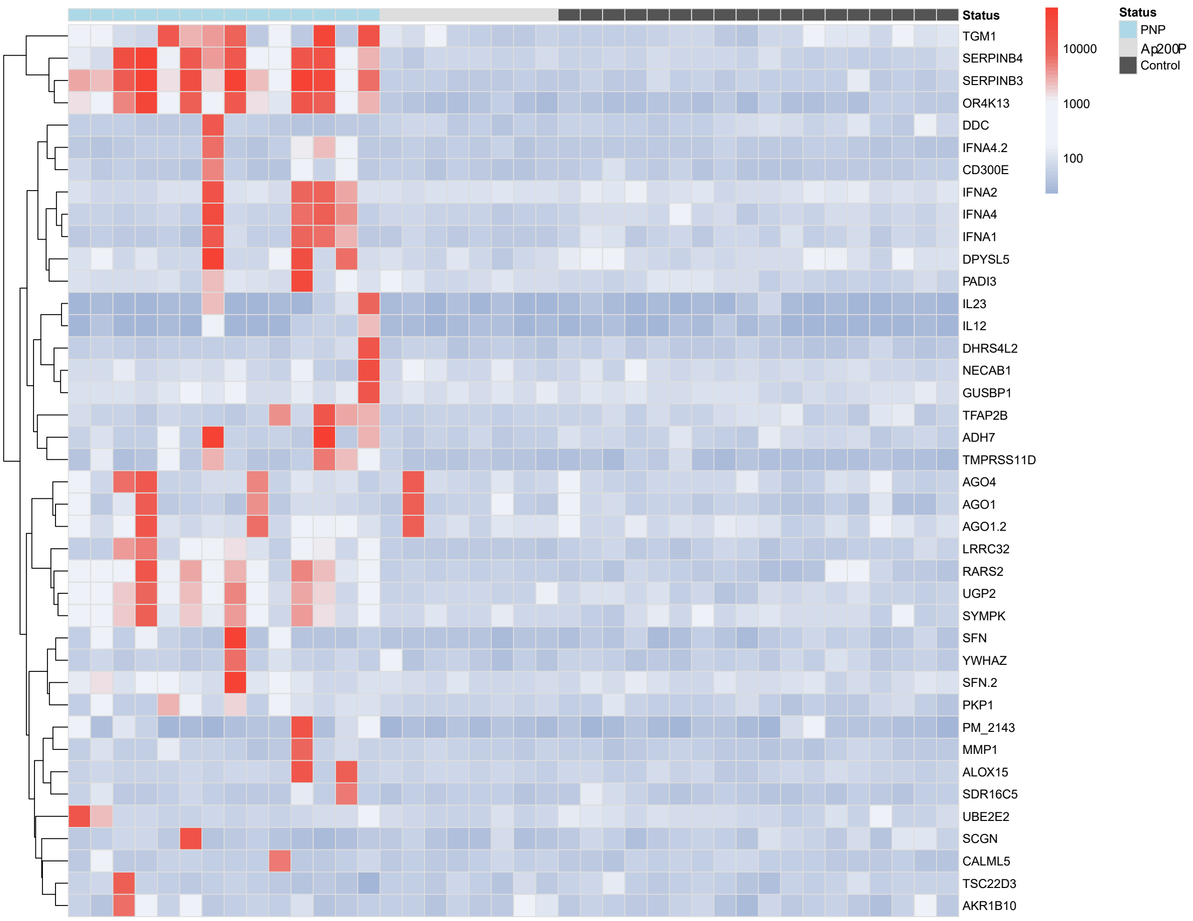

Figure S5 – Microarray signal intensities for top PNP-enriched proteins

The heatmap represents the intensities of autoantibody signals from the microarray screening within the discovery dataset. It highlights the 42 proteins exhibiting the most significant fold-change differences in autoantibody levels between paraneoplastic pemphigus cases and healthy controls. Results for Ap200P were not included in the case-control enrichment analysis that prioritized the top candidates; however, results for patients with Ap200P autoantibodies are provided for comparative purposes.

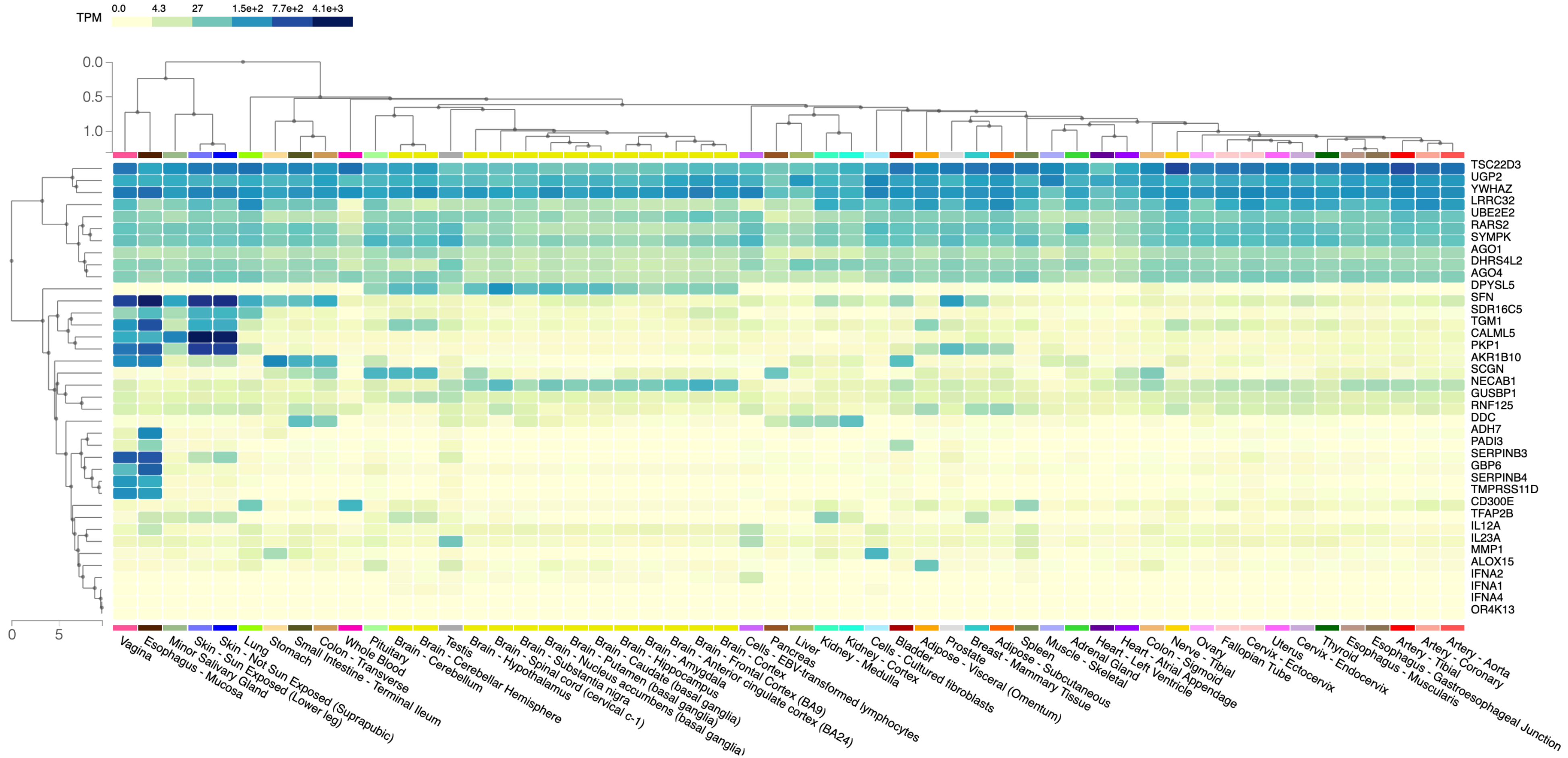

Figure S6 – Tissue expression of putative autoantigens (GTEx)

The figure shows gene expression data from GTEx by gene and tissue. The GTEx database was queried with putative autoantigens to explore their tissue specificity (GTEx Multi Gene Query: https://gtexportal.org/home/multiGeneQueryPage), Accessed Dec 5, 2022.

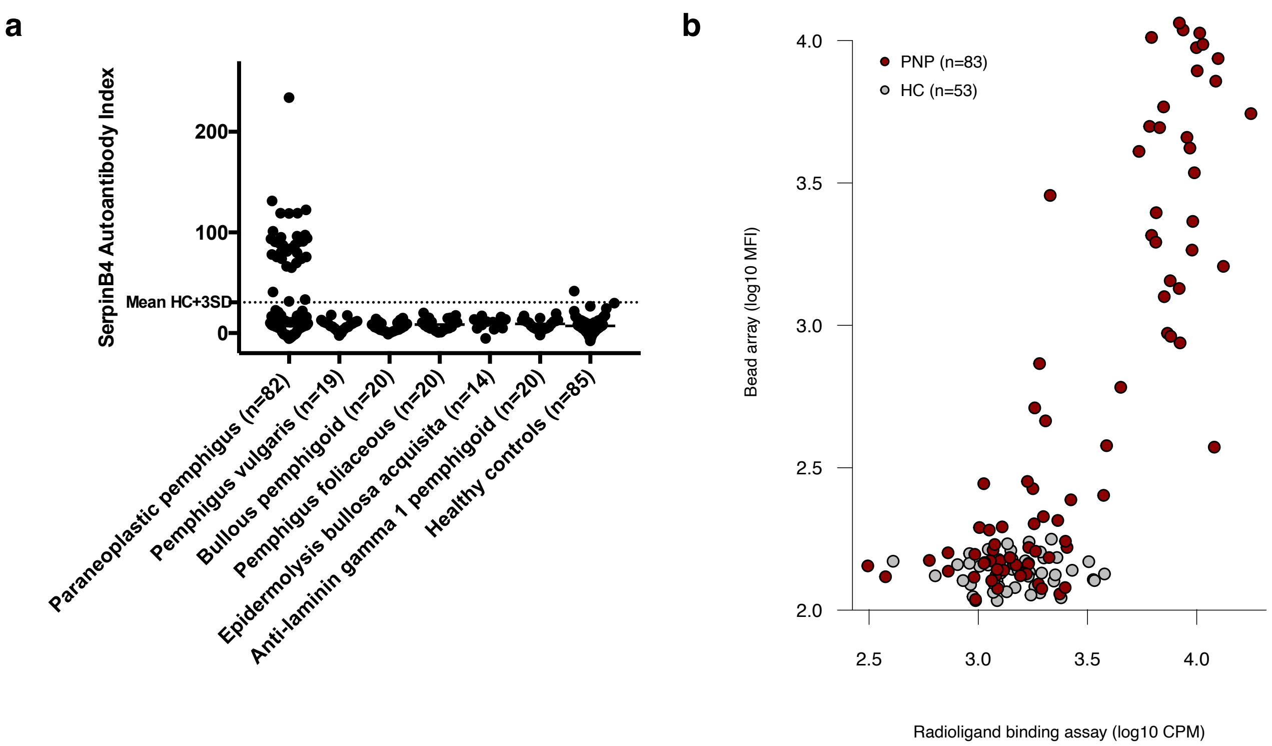

Figure S7 – Validation of SERPINB4 autoantibodies by radioligand binding assay

Autoantibodies against SERPINB4 were also detected with a radioligand binding assay using immunoprecipitation, a third independent method, specifically only in the paraneoplastic pemphigus cohort. The average signal in healthy controls plus three standard deviations (Mean HC + 3SD) was used as a cut-off the define the samples that were positive for autoantibodies against SERPINB4 (a). Correlation of autoantibody signal intensities in patients (PNP) and healthy controls (HC) obtained from the bead-array (y-axis) and the radioligand binding assay (x-axis) (b). MFI: mean fluorescence intensity; CPM: counts per minute.

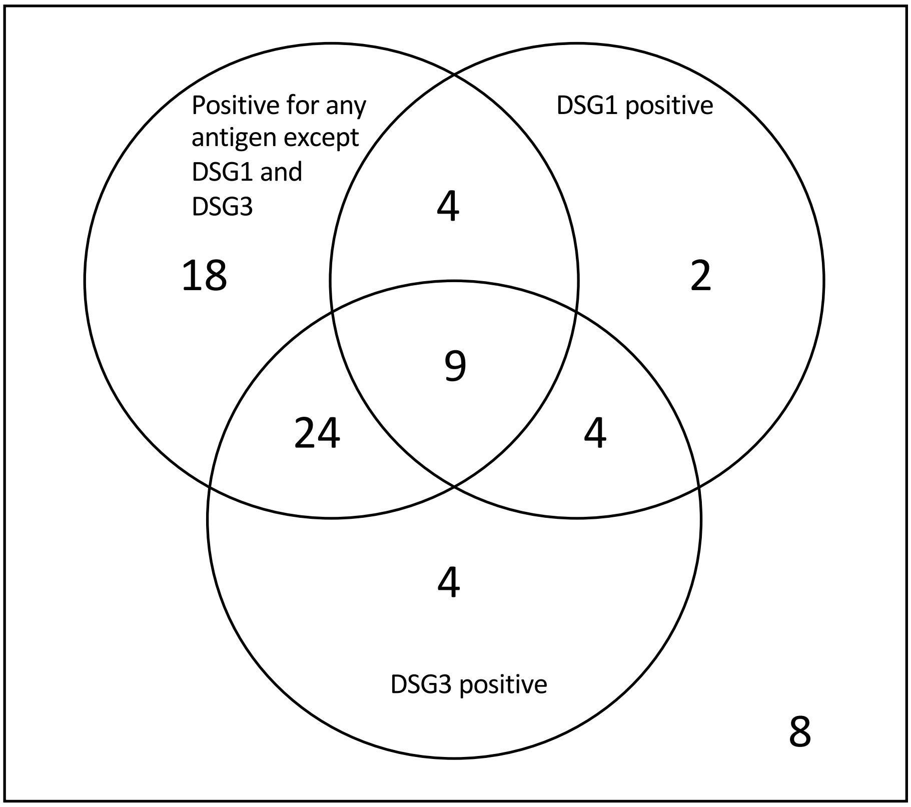

Figure S8 – PNP sensitivity: Desmogleins alone vs. combined biomarkers

Diagnostic performance of the new antigens in paraneoplastic pemphigus compared to the current clinical used desmogleins. The sensitivity of autoantibodies against desmoglein-1 (DSG1) and/or desmoglein-3 (DSG3) amounted to 64%, which increased to 89% when adding the new autoantigens. Eight patients diagnosed with PNP were negative for all three biomarker groups. Desmoglein data was missing for 11 patients with PNP.

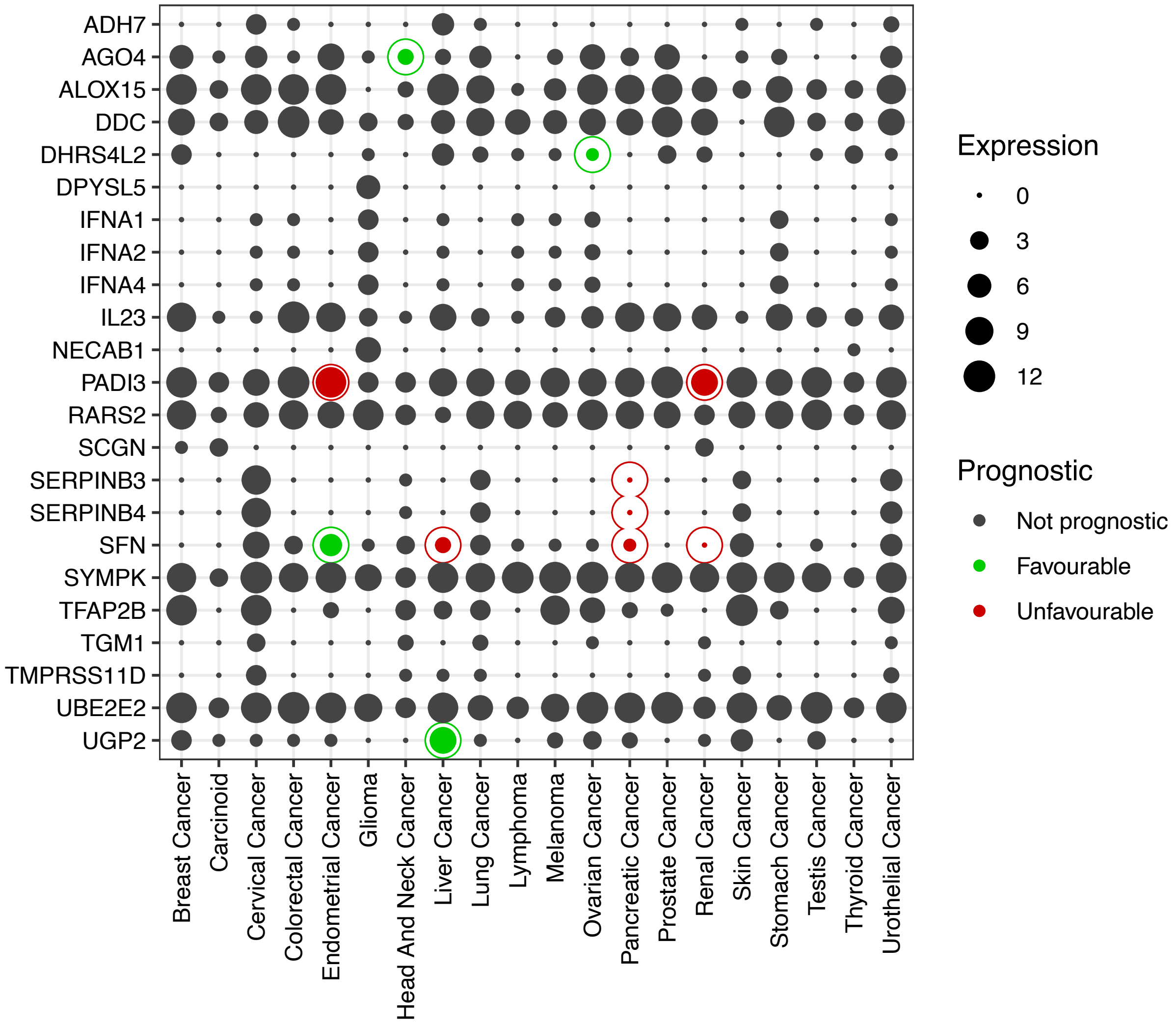

Figure S9 – Tumor expression and prognostic value of PNP autoantigens (HPA)

Tumor expression and prognostic relevance of candidate autoantigens in paraneoplastic pemphigus, according to the Human Protein Atlas (HPA) version 21.1. Prognostic value is based on correlations between mRNA expression levels and patient survival (Kaplan-Meier analysis). Expression levels are categorized as “Low,” “Medium,” or “High” by the HPA, based on immunohistochemistry performed on tumor tissue microarrays. This figure summarizes these categorical data for visualization purposes. For detailed expression and prognostic information, we refer readers to the original source (www.proteinatlas.org). Note that prognostic associations may occasionally appear for proteins with no detected expression by immunohistochemistry, potentially reflecting differences between RNA-seq and antibody-based measurements.

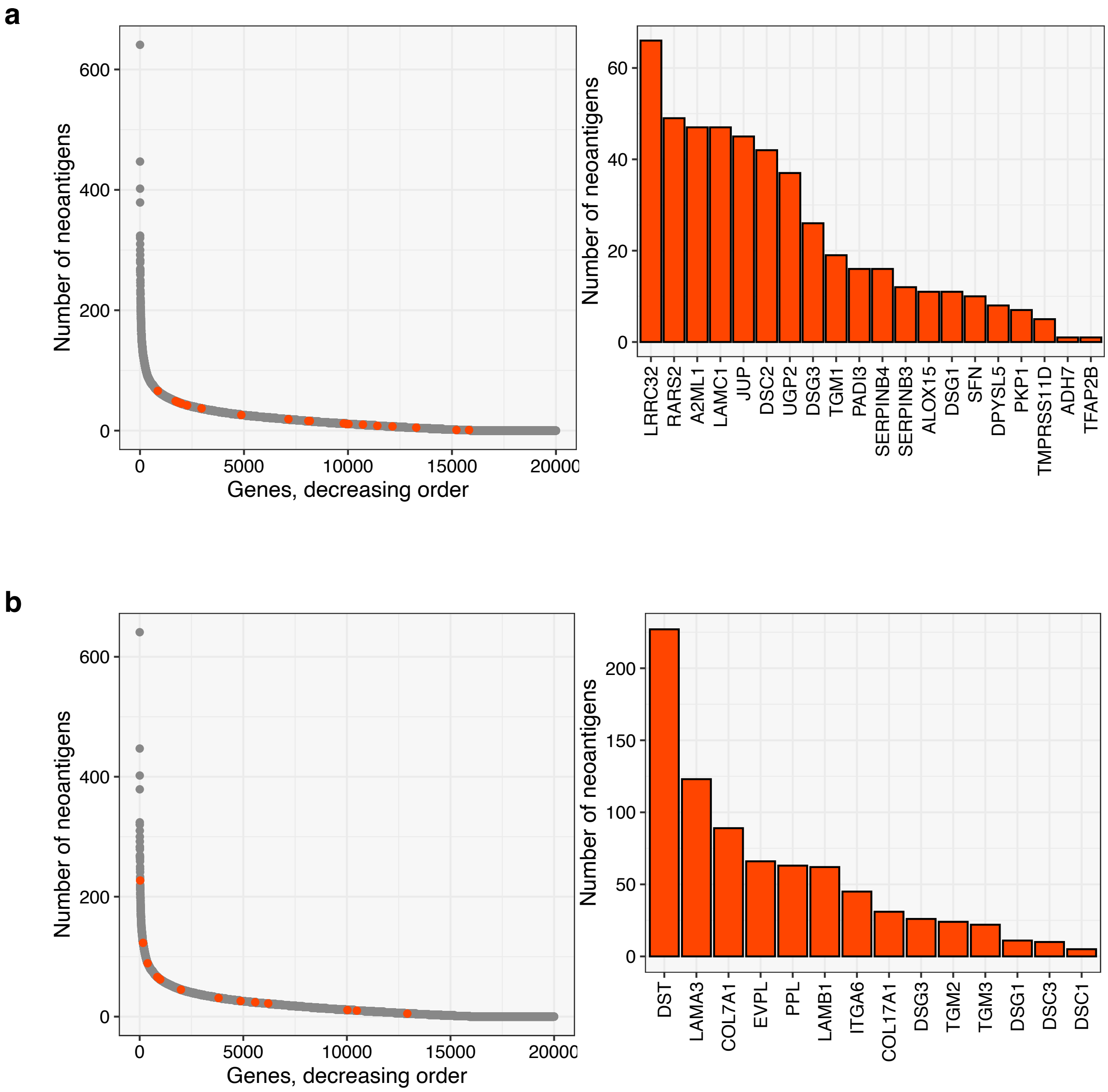

Figure S10 – Tumor neoantigen load for PNP and non-PNP autoantigens

The number of neoantigens registered in the Tumor-specific Neoantigen Database (2023) for autoantigens in paraneoplastic pemphigus (a), and non-paraneoplastic autoimmune blistering skin diseases (b). The leftmost panels display human genes ranked by their number of neoantigens (grey points), with the queried genes highlighted (red). The rightmost panels show the number of neoantigen calls in each of the genes. Autoantigens from the following non-paraneoplastic autoimmune skin diseases were included: Pemphigus vulgaris, Pemphigus Vegetans, Pemphigus foliaceus, Pemphigus herpetiformis, Anti-plakin dermatosis, Intercellular IgA dermatosis, Bullous pemphigoid, Mucous membrane pemphigoid, Epidermolysis bullosa acquisita, Anti-p200 pemphigoid, and Dermatitis herpetiformis.

Original article: TSNAdb v2.0: The Updated Version of Tumor-specific Neoantigen Database. Wu et al. Genomics, Proteomics & Bioinformatics

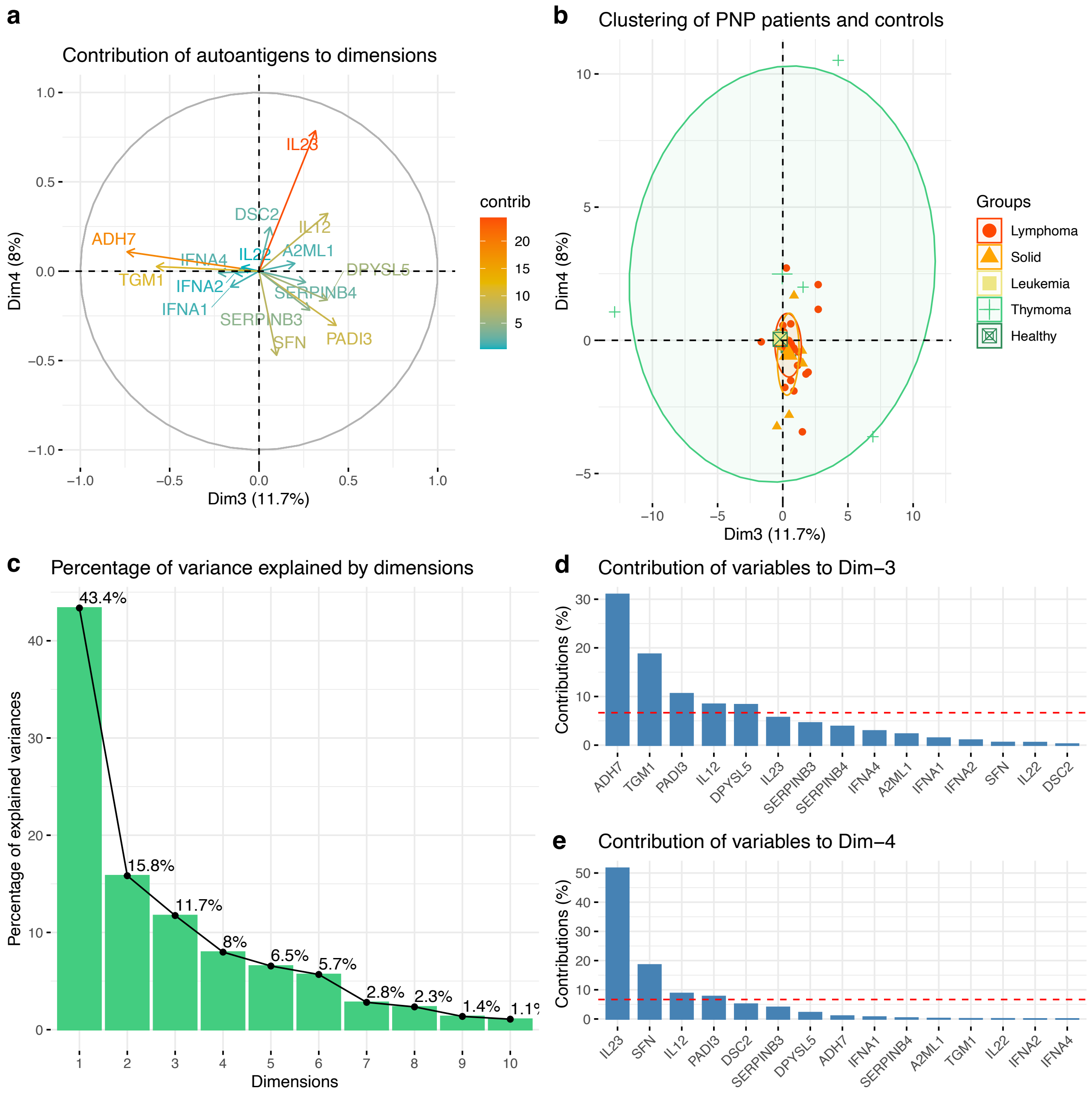

Figure S11 – PCA of autoantibody profiles (Dimensions 3 & 4)

The main axes (dimensions 3 and 4) in autoantibody reactivities calculated using principal component analysis of the bead-array signal intensities. (a) The arrows display the direction and size of the most important autoantibody contributions (contrib) in PCA space; dimensions 3 and 4. (b) Clusters of subjects with similar results. (c) Scree plot showing the variance explained by each component. (d) and (e) detail the contributing autoantigens in dimensions 3 and 4.

a

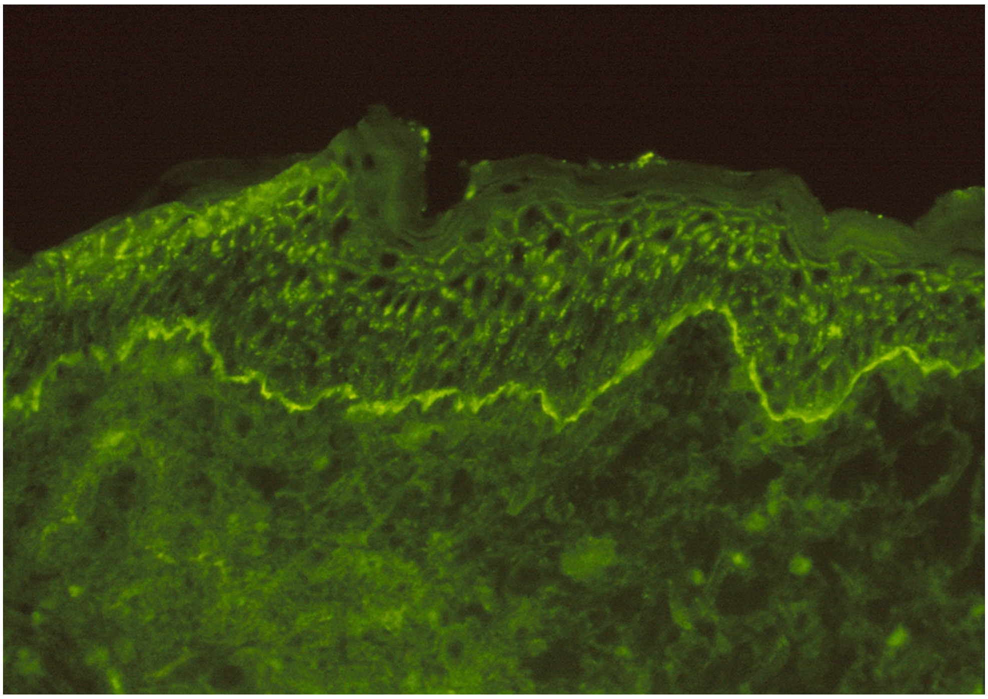

b

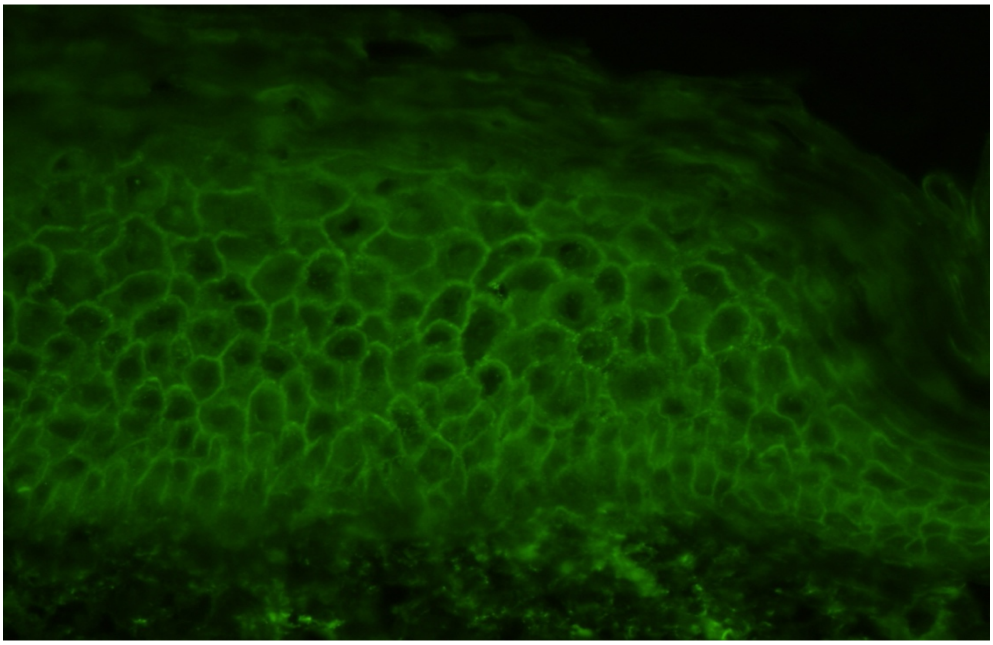

Figure S12 – Immunofluorescence for PNP diagnosis

Direct (a) and indirect (b) immunofluorescence images from a representative case diagnosed with PNP.

Supplementary Table S1 – Autoantibody profile in patients with PNP (n = 84)

	Autoantibody positive	Autoantibody negative
SERPINB3	49	35
SERPINB4	34	50
A2ML1	49	35
SFN	13	71
DSC2	1	83
TGM1	14	70
ADH7	1	83
PADI3	3	81
IL12	12	72
IL23	8	76
IL22	8	76
IFNA1	7	77
IL29	8	76
IL28B	4	80
IFNL4	4	80

Number of patients with PNP harbouring autoantibodies against PNP-specific autoantigens.

Supplementary Table S2– Optimal antigen sets for discriminating PNP cases

Number of auto-antigens	AUC	Antigen(s) with highest AUC
1	0.79	Same AUC in SerpinB3 and A2ML1
2	0.85	SerpinB3 and A2ML1
3	0.86	SerpinB3, A2ML1 and SFN
4	0.87	SerpinB3, A2ML1, SFN and IL29
**5**	**0.88**	**SerpinB3, A2ML1, SFN, IL29 and Desmocollin2**
6	0.88	SerpinB3, A2ML1, SFN, IL29, Desmocollin2 and IL22
7	0.88	SerpinB3, A2ML1, SFN, IL29, Desmocollin2, IL22 and SerpinB4 or TGM1
8	0.88	SerpinB3, A2ML1, SFN, IL29, Desmocollin2, IL22, SerpinB4 and TGM1
9	0.88	SerpinB3, A2ML1, SFN, IL29, Desmocollin2, IL22, SerpinB4, TGM1 and IFNA1
10	0.88	SerpinB3, A2ML1, SFN, IL29, Desmocollin2, IL22, SerpinB4, TGM1, IFNA1 and ADH7 or IL23 or IFNL4
11	0.88	SerpinB3, A2ML1, SFN, IL29, Desmocollin2, IL22, SerpinB4, TGM1, IFNA1 and ADH7/IL23 or ADH7/IFNL4 or IL23/IFNL4
12	0.88	SerpinB3, A2ML1, SFN, IL29, Desmocollin2, IL22, SerpinB4, TGM1, IFNA1, ADH7, IL23 and IFNL4
13	0.88	SerpinB3, A2ML1, SFN, IL29, Desmocollin2, IL22, SerpinB4, TGM1, IFNA1, ADH7, IL23, IFNL4 and IL12
14	0.88	SerpinB3, A2ML1, SFN, IL29, Desmocollin2, IL22, SerpinB4, TGM1, IFNA1, ADH7, IL23, IFNL4, IL12 and IL28b
15	0.88	SerpinB3, A2ML1, SFN, IL29, Desmocollin2, IL22, SerpinB4, TGM1, IFNA1, ADH7, IL23, IFNL4, IL12, IL28b and PADI3

The most optimal combination of antigens able to discern patients with PNP from other control cases and healthy blood donors was examined by the highest area under the curve (AUC) of a receiver operating characteristic curve (ROC). The highest AUC (0.88) was observed when at least 5 antigens were combined.

Supplementary Table S3 – Diagnostic performance of multi-antigen autoantibody panel

Number of auto-antigens	PNP	ALG1	BP	EBA	PV	PF	Cancer	Healthy
0	20	29	20	14	19	19	37	104
1	21	1	0	0	0	1	1	1
2	30	0	0	0	0	0	0	0
3	13	0	0	0	0	0	0	0
4	0	0	0	0	0	0	0	0
5	0	0	0	0	0	0	0	0
**Total**	**84**	**30**	**20**	**14**	**19**	**20**	**38**	**105**

Sensitivity: 76.2%
Specificity: 98.4%
PPV: 94.1%
NPV: 92.4%
FDR: 5.9%

Diagnostic performance of the most optimal autoantibody panel. An autoantibody score, or sum of the antigens for which individuals harbor antibodies to, was calculated to evaluate the performance of the new putative autoantigens. Autoantibodies against just one of the antigens in the autoantibody panel was enough to achieve high sensitivity and specificity. Around 50% of patients with PNP had autoantibodies against more than one autoantigen in the panel, showing their high specificity.

Supplementary Table S4 – Association of SERPINB3 autoantibodies to clinical disease manifestations

SERPINB3					
**Phenotype**	**Number of subjects**				**Fisher exact test P value**
**Diagnosed**	No	No	Yes	Yes	
**Autoantibodies**	Neg	Pos	Neg	Pos	
Diffuse large B cell lymphoma	32	48	3	1	0.3
Castleman disease	34	44	1	5	0.4
Follicular lymphoma	34	39	1	10	0.02
Thymoma	35	45	0	4	0.13
Lymphoma, not further specified	29	37	6	12	0.6
Bronchiolitis obliterans	5	1	3	14	0.0086

PNP (n=84). Diagnoses with less than 4 cases were not included in the analysis. Two-sided Fisher's exact test was used.

Supplementary Table S5 – Association of A2ML1 autoantibodies to clinical disease manifestations

A2ML1					
**Phenotype**	**Number of subjects**				**Fisher exact test P value**
**Diagnosed**	No	No	Yes	Yes	
**Autoantibodies**	Neg	Pos	Neg	Pos	
Diffuse large B cell lymphoma	32	48	3	1	0.3
Castleman disease	34	44	1	5	0.4
Follicular lymphoma	33	40	2	9	0.1
Thymoma	31	45	0	4	0.15
Lymphoma, not further specified	29	37	6	12	0.6
Bronchiolitis obliterans	6	2	2	15	0.0036

PNP (n=84). Diagnoses with less than 4 cases were not included in the analysis. Two-sided Fisher's exact test was used.

Supplementary Table S6 – Association of SERPINB4 autoantibodies to clinical disease manifestations

SERPINB4					
**Phenotype**	**Number of subjects**				**Fisher exact test P value**
**Diagnosed**	No	No	Yes	Yes	
**Autoantibodies**	Neg	Pos	Neg	Pos	
Diffuse large B cell lymphoma	46	34	4	0	0.14
Castleman disease	49	29	1	5	0.04
Follicular lymphoma	46	27	4	7	0.11
Thymoma	49	31	1	3	0.3
Lymphoma, not further specified	39	27	11	7	1
Bronchiolitis obliterans	6	2	5	12	0.08

PNP (n=84). Diagnoses with less than 4 cases were not included in the analysis. Two-sided Fisher's exact test was used.

Supplementary Table S7 – Association of IFNA1 autoantibodies to clinical disease manifestations

IFNA1					
**Phenotype**	**Number of subjects**				**Fisher exact test P value**
**Diagnosed**	No	No	Yes	Yes	
**Autoantibodies**	Neg	Pos	Neg	Pos	
Diffuse large B cell lymphoma	74	6	3	1	0.3
Castleman disease	71	7	6	0	1
Follicular lymphoma	66	7	11	0	0.59
Thymoma	76	4	1	3	0.0014
Lymphoma, not further specified	59	7	18	0	0.34
Bronchiolitis obliterans	6	0	17	0	*NA*

PNP (n=84). Diagnoses with less than 4 cases were not included in the analysis. Two-sided Fisher's exact test was used.

Supplementary Table S8 – Summary of clinical features of the PNP cohort (n = 84)

	All individuals (n = 84)	Autoantibody positive (n = 65)	Autoantibody negative (n = 19)	Fisher exact test p-value
Male : Female (unknown)	34 : 40 (10)	23 : 34 (8)	11 : 6 (2)	0.1
**Age (years)**				
10-30	4	4	0	0.57
31-60	29	24	5	0.58
61+	36	25	11	0.19
Unknown	15	12	3	1
**Bronchiolitis obliterans**	17	17	0	0.01
**Myasthenia gravis**	5	5	0	0.58
**Mucocutaneous lesions**				
Erythema	39	33	6	0.19
Blister	32	24	8	0.79
Erosion	24	20	4	0.57
Nail lesion	1	1	0	1
**Site of cutaneous lesions**				
Trunk	46	39	7	0.11
Extremities	40	32	8	0.61
Head	17	14	3	0.75
**Site of mucosal lesion**				
Oral	59	51	8	0.004
Ocular	26	25	1	0.005
Nasal	2	2	0	1
Genital	14	13	1	0.17
**Neoplasms**				
Follicular lymphoma	11	11	0	0.06
Diffuse B-cell lymphoma	4	4	0	0.57
Lymphoma, not further specified	18	15	3	0.75
Castleman's disease	6	6	0	0.33

	All individuals (n = 84)	Autoantibody positive (n = 65)	Autoantibody negative (n = 19)	Fisher exact test p-value
Thymoma	4	4	0	0.57
Chronic lymphocytic leukemia	1	0	1	0.23
Plasmacytoma	1	1	0	1
Follicular dendritic cell sarcoma	1	1	0	1
Rectal cancer	1	1	0	1
Gastrointestinal stromal cell tumour	1	1	0	1
Hepatocarcinoma	1	1	0	1
Myofibroblastoma	1	1	0	1
Gastric cancer	3	0	3	0.01
Thyroid cancer	1	1	0	1
Lung cancer	1	0	1	0.23
Mediastinal tumour	1	1	0	1
Intrapelvic tumour	1	1	0	1
Unknown	27	16	11	0.01

Two-sided Fisher's exact test was used and multiple comparison Bonferroni correction applied to each category: age, mucocutaneous lesions, site of cutaneous lesions, site of mucosal lesion and neoplasm.

Supplementary Note I – Recombinant proteins included in the bead-array experiment

For a full list of proteins analyzed in the same experiment see Le Voyer et al. (*Nature*, 2024).

ADH7 (OriGene, TP324304)
ALOX15 (OriGene, TP306621)
alpha 2 macroglobulin like (OriGene, TP319615)
anti-IgG (JacksonImmunoResearch, 309-005-082)
Desmocolin 2 (OriGene, TP309218)
Desmoglein-1 (Abcam, ab114443)
Desmoglein-3 (LifeSpan Biosciences, LSG137004)
DPYSL5 (OriGene, TP302631)
EBNA1 (Abcam, ab138345)
IFNA1 (Origene, TP721103; MedChemExpress, HY-P70241; Sigma, SRP4596)
IFNA10 (Origene, TP314055)
IFNA14 (Prospec, cyt-135)
IFNA16 (Bio-techne, 11190-1)
IFNA17 (Origene, TP320824)
IFNA2 (PBL Assay Science, PBL11101-2; Origene, TP321091)
IFNA21 (Origene, TP310115)
IFNA4 (Origene, TP323649)
IFNA5 (Origene, TP310825)
IFNA6 (Origene, TP760329)
IFNA7 (Bio-techne, 11079-IF)
IFNA8 (Origene, TP311169)
IFNB1 (MedChemExpress, HY-P73128)
IFNE (R&D Systems, 9667-ME/CF)
IFNG (MedChemExpress, HY-P7025; Origene, TP721239)
IFNK (Cusabio, CSB-EP889172HU)
IFNL4 (R&D Systems, 9165-IF)
IFNW1 (MedChemExpress, HY-P7201; Origene, TP721113)
IL12 (Miltenyi, 130-129-718).
IL17A (Origene, TP318057)
IL17F (MedChemExpress, HY-P70540; Origene, TP723203)
IL22 (Origene, TP309995; Miltenyi, 130-096-297)
IL23 (MedChemExpress, HY-P73193)
IL28a (Sino Biological, 12340-H0By)
IL28b (Abcam, ab276441)
IL29 (Abcam, ab155625)
IL6 (Origene, DA3547; MedChemExpress, HY-P7044G)
JUP (Abnova, H00003728-P01)
Laminin gamma 1 (OriGene, TP316928)
LRRC32 (R&D Systems, 6055-LR)
OR4K13 (Bio-techne, H00390433-P01)
PADI3 (Aviva Systems, OPCA03266)
PKP1 (OriGene, TP316972)
RARS2 (Bio-techne, H00057038-P01)
SERPINB3 (OriGene, TP302683)
SERPINB4 (OriGene, TP790064)
SFN (OriGene, TP304045)
TFAP2B (Bio-techne, H00007021-P01)
TGM1 (OriGene, TP304492)
TMPRSS11D (Abnova, H00009407)
UGP2 (OriGene, TP308376)

Supplementary Note II – List of PNP autoantigens identified with protein microarrays

ADH7	alcohol dehydrogenase 7
AGO1	argonaute RISC component 1
AGO4	argonaute RISC component 4
AKR1B10	aldo-keto reductase family 1 member B10
ALOX15	arachidonate 15-lipoxygenase
CALML5	calmodulin like 5
CD300E	CD300e molecule
DDC	dopa decarboxylase
DHRS4L2	dehydrogenase/reductase 4 like 2
DPYSL5	dihydropyrimidinase like 5
GBP6	guanylate binding protein family member 6
GUSBP1	GUSB pseudogene 1
IFNA1	interferon alpha 1
IFNA2	interferon alpha 2
IFNA4	interferon alpha 4
IL12	interleukin 12
IL23	Interleukin 23
LRRC32	leucine rich repeat containing 32
MMP1	matrix metallopeptidase 1
NECAB1	N-terminal EF-hand calcium binding protein 1
OR4K13	olfactory receptor family 4 subfamily K member 13
PADI3	peptidyl arginine deiminase 3
PKP1	plakophilin 1
PM_2143	centromere protein B
RARS2	arginyl-tRNA synthetase 2
RNF125	ring finger protein 125
SCGN	secretagogin
SDR16C5	short chain dehydrogenase/reductase family 16C member 5
SERPINB3	serpin family B member 3
SERPINB4	serpin family B member 4
SFN	stratifin
SYMPK	symplekin
TFAP2B	transcription factor AP-2 beta
TGM1	transglutaminase 1
TMPRSS11D	transmembrane serine protease 11D
TSC22D3	TSC22 domain family member 3
UBE2E2	ubiquitin conjugating enzyme E2 E2
UGP2	UDP-glucose pyrophosphorylase 2
YWHAZ	tyrosine 3-monooxygenase/tryptophan 5-monooxygenase activation protein zeta
